# Cross-Resistance between Cry1 Proteins in Fall Armyworm (*Spodoptera frugiperda*) May Affect the Durability of Current Pyramided Bt Maize Hybrids in Brazil

**DOI:** 10.1371/journal.pone.0140130

**Published:** 2015-10-16

**Authors:** Daniel Bernardi, Eloisa Salmeron, Renato Jun Horikoshi, Oderlei Bernardi, Patrick Marques Dourado, Renato Assis Carvalho, Samuel Martinelli, Graham P. Head, Celso Omoto

**Affiliations:** 1 Department of Entomology and Acarology, Escola Superior de Agricultura “Luiz de Queiroz” (ESALQ/USP), Piracicaba, São Paulo, Brazil; 2 Monsanto do Brasil Ltda, São Paulo, São Paulo, Brazil; 3 Monsanto LLC, Saint Louis, Missouri, United States of America; University of Tennessee, UNITED STATES

## Abstract

Genetically modified plants expressing insecticidal proteins from *Bacillus thuringiensis* (Bt) offer valuable options for managing insect pests with considerable environmental and economic benefits. Despite the benefits provided by Bt crops, the continuous expression of these insecticidal proteins imposes strong selection for resistance in target pest populations. Bt maize (*Zea mays*) hybrids have been successful in controlling fall armyworm (*Spodoptera frugiperda*), the main maize pest in Brazil since 2008; however, field-evolved resistance to the protein Cry1F has recently been reported. Therefore it is important to assess the possibility of cross-resistance between Cry1F and other Cry proteins expressed in Bt maize hybrids. In this study, an F_2_ screen followed by subsequent selection on MON 89034 maize was used to select an *S*. *frugiperda* strain (RR) able to survive on the Bt maize event MON 89034, which expresses the Cry1A.105 and Cry2Ab2 proteins. Field-collected insects from maize expressing the Cry1F protein (event TC1507) represented most of the positive (resistance allele-containing) (iso)families found. The RR strain showed high levels of resistance to Cry1F, which apparently also conferred high levels of cross resistance to Cry1A.105 and Cry1Ab, but had only low-level (10-fold) resistance to Cry2Ab2. Life history studies to investigate fitness costs associated with the resistance in RR strain revealed only small reductions in reproductive rate when compared to susceptible and heterozygous strains, but the RR strain produced 32.2% and 28.4% fewer females from each female relative to the SS and RS (pooled) strains, respectively. Consistent with the lack of significant resistance to Cry2Ab2, MON 89034 maize in combination with appropriate management practices continues to provide effective control of *S*. *frugiperda* in Brazil. Nevertheless, the occurrence of Cry1F resistance in *S*. *frugiperda* across Brazil, and the cross-resistance to Cry1Ab and Cry1A.105, indicates that current Cry1-based maize hybrids face a challenge in managing *S*. *frugiperda* in Brazil and highlights the importance of effective insect resistance management for these technologies.

## Introduction

Agriculture in tropical regions poses a number of challenges relative to temperate zones primarily due to the longer growing seasons and absence of freezing winter temperatures [1]. Consequently, effectively managing pest insect populations is one the most important requirements for protecting crop yields in tropical environments [2, 3]. The availability of Bt plants engineered to produce insecticidal proteins from the bacterium *Bacillus thuringiensis* (Bt) has provided valuable options for managing certain insect pests with considerable environmental and economic benefits [4]. The area planted with Bt crops was approximately 78.8 M ha worldwide in 2014 [5]. Benefits of Bt crops include effective management of target pests [6, 7], decreased use of synthetic insecticides [8–10], and reduced harm to non-target organisms [7, 11, 12]. Brazil is classified as a mega-diverse country because of its extensive biodiversity [13] and is one of the world’s leading agricultural producers [14]. Consolidation of Brazilian agriculture has occurred through the use of science-based technologies, which has led to significant productivity gains [15]. For instance, Bt maize (*Zea mays*) hybrids have been approved for cultivation in Brazil since 2007 [16]. The adoption of these crops reached 80% of the maize-growing area in Brazil during the 2013–2014 cropping season [17], largely with event TC1507, a single-mode-of-action Bt maize event expressing the Cry1F protein.

The fall armyworm, *Spodoptera frugiperda* (J.E. Smith) (Lepidoptera: Noctuidae), is the most important lepidopteran pest of maize in Brazil [18]. Historically, control of the fall armyworm had relied largely on the use of synthetic insecticides, leading to the evolution of resistance to most of those chemicals [19, 20]. Bt maize hybrids have been useful in controlling *S*. *frugiperda* in Brazil [21–23]. Despite the economic benefits provided by Bt crops [24], the continuous expression of *cry* genes in Bt plants imposes a strong selection pressure for resistance in target pest populations [25], and the benefits of Bt crops would be reduced when insect pests evolve resistance [26]. Field-evolved resistance to Bt crops has been documented in *Busseola fusca* (Füller) resistant to Cry1Ab maize in South Africa [27], in *S*. *frugiperda* resistant to Cry1F maize in Puerto Rico and Brazil [28, 29], in *Pectinophora gossypiella* (Saunders) resistant to Cry1Ac cotton in India [30], and in *Diabrotica virgifera virgifera* LeConte resistant to Cry3Bb1 maize in the USA [31].

The evolution of resistance of *S*. *frugiperda* in Brazil to the proteins expressed in Bt maize technologies is a concern because of Brazil’s intense agricultural production systems with multiple cropping seasons [32] and low compliance with non-Bt structured refuge recommendations [33]. Resistance of *S*. *frugiperda* to Cry1F in Brazil was confirmed by Farias et al. [29, 33], after several years of anecdotal reports. In addition, several recent publications suggest that there is a low level of cross-resistance between Cry1F and other Cry1 proteins [34–38]. In this paper, we characterize cross-resistance between Cry1F and other Cry proteins in *S*. *frugiperda*.

## Results and Discussion

### Selection and characterization of resistance

An F_2_ screen [39] was utilized to identify *S*. *frugiperda* larvae capable of surviving on MON 89034. Fall armyworm field populations were sampled across Brazil in 2012. Out of 552 two-parent families tested, the 155 sampled from the state of Bahia were the source of most of the positive (iso)families found (Table 1; S1 Table). Insects collected on maize expressing Cry1F protein (population BA27) resulted in 41 positive (resistance alleles-containing) (iso)families identified by testing on MON 89034 leaf tissues. The number of families was 2.4 times that found in other field-collected *S*. *frugiperda* populations collected from non-Bt maize in the same geographic region (BA31). Insects collected in the state of Goiás represented the second-highest number of positive families (8/130), followed by Mato Grosso do Sul (5/99), Paraná (4/108) and Mato Grosso (0/60). Considering only the *S*. *frugiperda* larvae collected on non-Bt maize, 34 positive (iso)families were identified from the 453 tested.

**Table 1:** Number of two-parent families tested and positive lines of *S*. *frugiperda* (those with any offspring that survived on MON 89034 maize) identified using F_2_ screen method.

Insect population	County/State	Year	Host source	F_2_ lines tested	Positive F_2_ lines
BA27	São Desidério, BA	2012	Cry1F maize	99	41
BA31	Luís Eduardo Magalhães, BA	2012	non-Bt maize	56	17
GO22	Montividiu, GO	2012	non-Bt maize	74	7
GO23	Caiapônia, GO	2012	non-Bt maize	56	1
MT19	Sinop, MT	2012	non-Bt maize	26	0
MT20	Campo Novo do Parecis, MT	2012	non-Bt maize	34	0
MS11	São Gabriel do Oeste, MS	2012	non-Bt maize	51	4
MS12	Chapadão do Sul, MS	2012	non-Bt maize	21	1
MS13	Dourados, MS	2012	non-Bt maize	27	0
PR34	Sabáudia, PR	2012	non-Bt maize	69	4
PR38	Campo Mourão, PR	2012	non-Bt maize	39	0
Total	-			552	75

The BA27 population of *S*. *frugiperda* had also been assessed for Cry1F resistance through an F_2_ screen using purified Cry1F protein, and the results confirmed the presence of Cry1F resistance alleles [33]. The results of the F_2_ screen indicated a higher rate of positive (iso)familes derived from BA27,collected from Cry1F maize and known to contain Cry1F resistance alleles [33], compared to BA31 (collected from non-Bt maize). These results indicated the potential for cross-resistance in *S*. *frugiperda* between Cry1F and Cry1A.105, one of the two Bt proteins expressed by MON 89034 maize. Cry1A.105 is a chimeric protein with domains I and II and the C-terminal from Cry1Ac, and domain III almost identical to Cry1F [34], and this similarity in amino acid sequence between Cry1F and Cry1A.105 can explain the apparent cross-resistance [37].

After screening the BA27 population against MON 89034 plants, surviving larvae were recovered and reared on MON 89034 leaves for 15 consecutive generations to establish a resistant strain (RR). This strain was used in reciprocal crosses with a laboratory-raised susceptible strain (SS) to generate two heterozygous strains (S♂R♀ and S♀R♂). In greenhouse tests, survival of the RR strain was high when fed on MON 89034 plants relative to non-Bt plants (Table 2, S2 Table). None of the other *S*. *frugiperda* strains tested (SS, S♂R♀, S♀R♂) survived on MON 89034 leaf tissue, indicating that the resistance trait in the RR strain was functionally recessive (Table 2; S2 Table). All four strains (SS, S♂R♀, S♀R♂, RR) showed high survival rates (>70%) on non-Bt maize leaf tissue (Table 2; S2 Table).

**Table 2:** Survival of *S*. *frugiperda* larvae per plant (mean ± SE) on MON 89034 maize and non-Bt near-isoline in greenhouse trials.

FAW strain	Host	Surviving larvae^a^
RR	MON 89034	0.75 ± 0.04 a
	Non-Bt maize	0.80 ± 0.04 a
S♂R♀	MON 89034	0.00 ± 0.00 b
	Non-Bt maize	0.80 ± 0.04 a
S♀R♂	MON 89034	0.00 ± 0.00 b
	Non-Bt maize	0.85 ± 0.04 a
SS	MON 89034	0.00 ± 0.00 b
	Non-Bt maize	0.75 ± 0.04 a

^a^ Values followed by the same letter are not significantly different (LSD *t*-test. *P* > 0.05).

**Fig 1:**
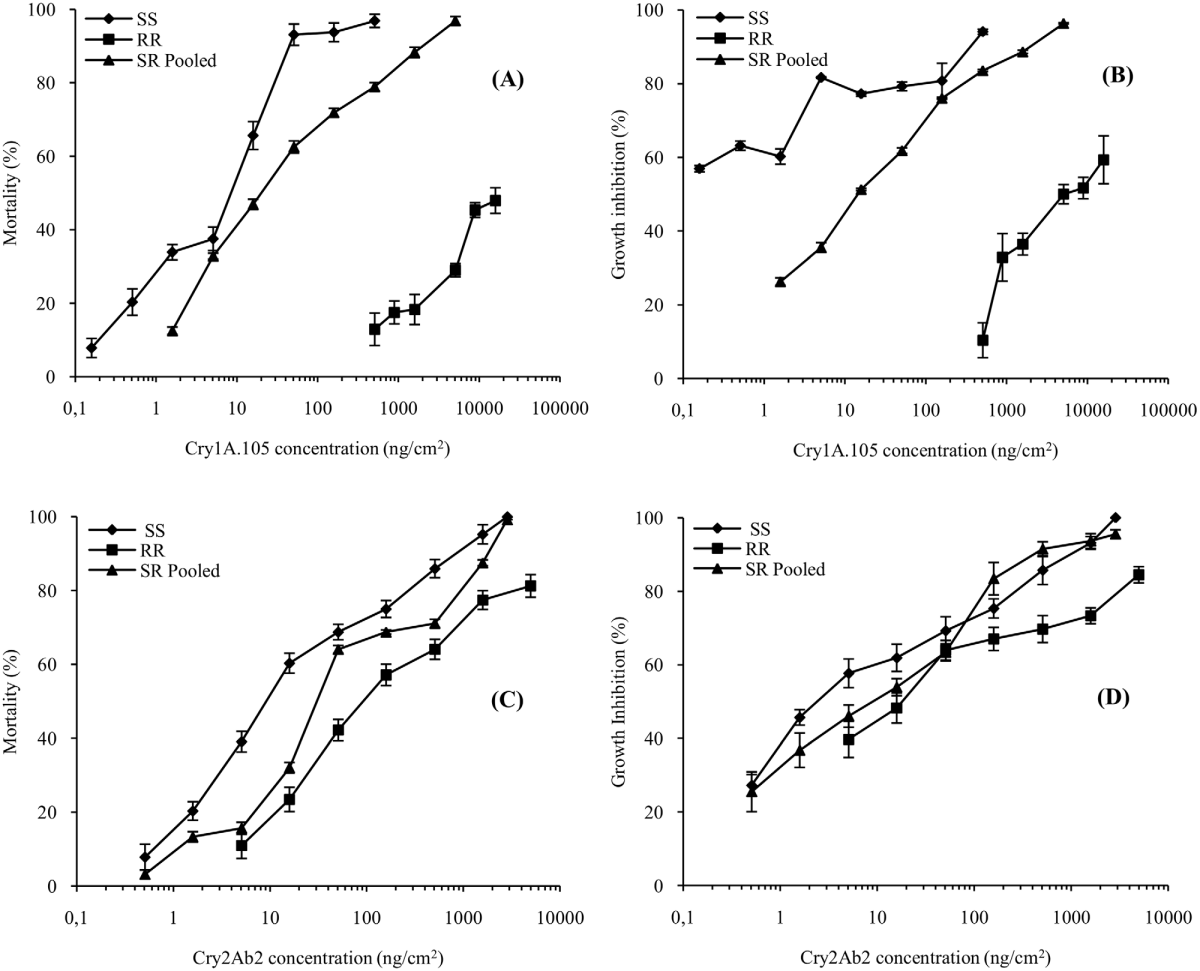
Concentration-response of *S*. *frugiperda* in diet-overlay bioassays with purified Cry1A.105 (A and B) and Cry2Ab2 (C and D) proteins. Each data point represents the mean of four replicates, corrected for control mortality. Error bars represent SD.

To understand the resistance patterns observed in the F_2_ screen on MON 89034 leaf tissue, the RR strain was characterized using diet-overlay bioassays with purified Cry1A.105 and Cry2Ab2 proteins (Table 3; S3 Table). The RR strain had more than 3,300-fold resistance to Cry1A.105 (MIC_50_ > 16,000 ng/cm^2^) relative to the SS strain. In contrast, a low level of resistance (10-fold) was observed for Cry2Ab2 relative to the SS strain (Table 3; S3 Table), which may only have reflected the greater vigor of the field-derived RR strain compared with the laboratory-reared SS strain. The MIC_50_, a concentration that inhibits 50% of larvae from molting to second instar after 7 days, for Cry1A.105 tested against the RR strain could not be precisely determined because 50% mortality was never achieved, even at the highest concentration (Fig 1; S4 Table). Using a Cry1A.105 stock solution of 1,000 μg/ml, it was possible to generate a maximum concentration of 16,000 ng/cm^2^ of diet in the overlay bioassay; consequently, the MIC_50_ for Cry1A.105 must be greater than 16,000 ng/cm^2^. The resistance ratio of the RR strain tested on Cry1A.105 was much lower when based on larval growth inhibition (EC_50_) (207-fold), indicating some level of Cry1A.105 activity against the RR strain (Table 3; Fig 1). Both heterozygous strains (reciprocal crosses) showed similar MIC_50_ values, ranging from 25.05 to 32.62 ng/cm^2^ for Cry1A.105 and from 45.63 to 48.35 ng/cm^2^ for Cry2Ab2 (Table 3; S3 Table). The MIC_50_ and EC_50_ of both heterozygous strains with Cry1A.105 were significantly lower than those of the RR strain, and higher than those of the SS strain (Table 3; S3 Table). With the exception of the EC values for Cry1A.105, the overlapping confidence limits for the MIC and EC values of the reciprocal F_1_ crosses between the SS and RR strains (S♂R♀, S♀R♂) indicated autosomal inheritance of the resistance trait in the RR strain.

**Table 3:** Concentration-response and growth inhibition response (MIC_50_ and EC_50_; ng/cm^2^) of *S*. *frugiperda* in diet-overlay bioassays with purified Cry1A.105 and Cry2Ab2 proteins.

FAW strain	*n*	Slope ± SE	MIC_50_ (95% IC)^a^	χ2(df)^b^	Resistance Ratio^c^	EC_50_ (95% CI)^d^	Resistance Ratio^c^
**Tests with Cry1A.105 protein**							
RR	384	-	> 16,000	-	> 3368	138.40 (102.21–191.02)	206.56
S♂R♀	502	1.14 ± 0.11	25.05 (18.80–32.06)	5.08 (6)	5.27	8.80 (5.96–12.80)	13.13
S♀R♂	445	1.42 ± 0.12	32.62 (23.98–47.67)	6.14 (6)	6.86	18.37 (15.87–29.38)	27.41
SR Pooled	947	1.16 ± 0.04	28.97 (20.53–39.90)	8.53 (6)	5.99	13.18 (12.39–13.97)	19.7
SS	448	1.20 ± 0.19	4.75 (2.67–7.04)	8.63 (4)	-	0.67 (0.53–0.84)	-
**Tests with Cry2Ab2 protein**							
RR	540	0.96 ± 0.09	146.96 (83.37–330.35)	16.83 (5)	10.44	19.05 (10.77–35.56)	10.82
S♂R♀	448	1.51 ± 0.12	48.35 (26.17–90.48)	18.07 (7)	3.43	16.01 (12.72–24.36)	9.09
S♀R♂	512	1.07 ± 0.12	45.63 (28.48–76.28)	17.24 (7)	3.24	13.51 (8.68–20.53)	7.67
SR Pooled	960	1.40 ± 0.10	45.36 (25.81–76.61)	8.53 (6)	3.10	13.52 (11.53–15.51)	7.68
SS	576	1.27 ± 0.09	14.06 (11.64–16.96)	7.63 (7)	-	1.76 (1.23–2.49)	-

^a^ MIC_50_: Concentration that inhibits molting to second instar in 50% of individuals after 7 days.

^b^ *P* > 0.05 in the goodness-of-fit test.

^c^ Resistance Ratio = (MIC_50_ or EC_50_ of indicated strain)/(MIC_50_ or EC_50_ of SS strain).

^d^ EC_50_: Effective concentration of protein required to cause 50% growth inhibition at 7 days.

Because we found no sex linkage or maternal effects associated with the inheritance of the resistance, the F_1_ data were pooled. Fig 1 shows the Cry1A.105 and Cry2Ab2 concentration-response curves for the three genotypes. There were differences in mortality and growth inhibition, and the SR pooled strain was significantly more sensitive than the RR colony to Cry1A.105 based on a comparison of confidence intervals for the EC_50_ and MIC_50_ (Table 3; Fig 1A and 1B). Nonetheless, the RR strain showed some response to Cry1A.105 (Fig 1A and 1B). Despite the approximately 10-fold resistance to Cry2Ab2 detected in the RR strain, the results of the concentration-response bioassay indicated that the SS, SR pooled and RR colonies had similar responses (mortality and growth inhibition) to Cry2Ab2 (Fig 1C and 1D).

The estimation of effective dominance (*D*_GIL_) defined by Bourguet et al. [40] generates values between 0 and 1, which indicate a range of responses from fully recessive to fully dominant, respectively. The evaluation of resistance in the RR strain based on growth inhibition at 508.7 ng of Cry1A.105 /cm^2^ indicated highly recessive resistance. The growth inhibition of susceptible (SS) and heterozygous strains (SR pooled) was 94.1% and 83.6%, respectively, when exposed to Cry1A.105 (508.7 ng/cm^2^ of diet), and 93.1% and 93.7%, respectively, when exposed to Cry2Ab2 at 1,589.8 ng/cm^2^ of diet (Table 4; S3 Table). The growth inhibition observed in RR neonates at the same concentrations of these two proteins was 10.3% and 73.3%, respectively (Table 4; S3 Table). Larval survival in controls (without Cry1A.105 and Cry2Ab2 protein) was 100% for all genotypes tested (data not shown). Overall, the results indicated that the resistance expressed in the RR strain is recessive.

**Table 4:** Estimates of effective dominance, *D*_GIL_ (after [40]), for the Cry1A.105 and Cry2Ab2 resistance traits in the RR *S*. *frugiperda* strain compared with the laboratory-reared SS strain, based on growth inhibition at 508.7 and 1589.8 ng of Cry1A.105 or Cry2Ab2/cm^2^ of diet, respectively.

Strain	SS (%)	RR (%)	SR Pooled (%)	Dominance
Cry1A.105 protein				
Mean growth inhibition (SD)	94.1 (0.7)	10.3 (4.7)	83.6 (0.3)	*D*_GIL_ = 0.13
Cry2Ab2 protein				
Mean growth inhibition (SD)	93.1 (1.7)	73.3 (2.2)	93.7 (2.0)	*D*_GIL_ = 0.03

### Cross-resistance between Cry1 proteins—*in planta* assays

The RR strain was used in leaf disc bioassays to test for cross-resistance among the Cry proteins expressed in Bt maize commercial hybrids and experimental lines: Cry1Ab (MON 810), Cry1F (TC1507), Cry1A.105 (Cry1A-P), Cry2Ab2 (Cry2A-P) and non-Bt maize. In 5-day leaf disc bioassays, all of the Bt maize showed complete control of the susceptible population (SS) with exception of the Cry1Ab-expressing event (MON 810), which had 61.6% larval survival (Table 5; S5 Table). This result was expected due to the known lower activity of Cry1Ab (MON 810) against *S*. *frugiperda* [23]. The maize products expressing Cry1F (TC1507) and Cry1Ab (MON 810) proteins had no effect on the survival or growth of the RR strain (Table 5; S5 Table). Despite allowing relatively high survival of the RR strain (80%), the Cry1A-P maize (expressing Cry1A.105) caused significant larval growth inhibition (76%) of RR larvae when compared to the non-Bt maize (Table 5, S5 Table). In contrast, Cry2A-P (expressing Cry2Ab2) produced near complete mortality of both RR and SS strains. Our results indicated significant levels of cross-resistance between Cry1F, Cry1Ab and Cry1A.105 in *S*. *frugiperda*, but not between the Cry1 and Cry2Ab2 proteins. Based on the gene sequences, the overall amino acid sequence identity of Cry1A.105 to Cry1Ab and Cry1F is 90.0% and 76.7%, respectively, which explains the cross-resistance observed among Cry1F, Cry1Ab and Cry1A.105 [41]. The potential for cross-resistance among the Cry1Ab/Ac, Cry1A.105 and Cry1F proteins in *S*. *frugiperda* through the alteration of shared binding sites was highlighted by Hernández-Rodríguez et al. [34]. In contrast, Cry2Ab2 has a distinct mode of action from that of Cry1F and Cry1A proteins [42] and therefore cross-resistance between Cry2Ab2 and Cry1F or Cry1A proteins is unlikely [34, 43]. Low levels of cross-resistance between Cry1F and Cry1A.105 also were detected in a *S*. *frugiperda* Cry1F-resistant strain isolated through an F_2_ screen from a field population sampled in south Florida, USA [35, 37, 44].

**Table 5:** Survival of *S*. *frugiperda* on leaf discs of different Bt and non-Bt maize plants.

Entry	Survival (%)	Larval stage of survivors			Weight (mg)^a^	WR (%)^b^
		L1	L2	L3		
**RR strain**						
Cry1A-P	80.0 ± 8.6 b	90.6 ± 5.8	9.4 ± 5.8	0.0 ± 0.0	1.9 ± 0.3	75.7
Cry2A-P	1.6 ± 1.6 c	100.0 ± 0.0	0.0 ± 0.0	0.0 ± 0.0	n.a	n.a
TC 1507 (Cry1F)	98.4 ± 1.6 a	0.0 ± 0.0	7.0 ± 3.4	93.0 ± 3.4	8.0 ± 1.2	0.0
MON 810 (Cry1Ab)	98.4 ± 1.6 a	0.0 ± 0.0	0.0 ± 0.0	100.0 ± 0.0	8.8 ± 0.4	0.0
Non-Bt maize	100.0 ± 0.0 a	0.0 ± 0.0	1.7 ± 1.7	98.3 ± 1.7	7.6 ± 2.6	n.a
**SS strain**						
Cry1A-P	0.0 ± 0.0 c	n.a	n.a	n.a	n.a	n.a
Cry2A-P	0.0 ± 0.0 c	n.a	n.a	n.a	n.a	n.a
TC 1507 (Cry1F)	0.0 ± 0.0 c	n.a	n.a	n.a	n.a	n.a
MON 810 (Cry1Ab)	61.6 ± 4.3 b	0.0 ± 0.0	13.8 ± 4.6	86.2 ± 4.6	4.3 ± 0.4	40.5
Non-Bt maize	90.0 ± 4.9 a	0.0 ± 0.0	8.4 ± 5.2	91.6 ± 5.2	7.2 ± 0.5	n.a

Values represent means ± SE. A separate ANOVA (Tukey’s test, *P*≤0.05) was conducted for treatments within each column (means followed by the same letter in column are not significantly different).

^a^ Mean weight of survivors.

^b^ Weight reduction compared to control (non-Bt).

n.a. Not applicable

### Fitness cost of resistance

**Fig 2:**
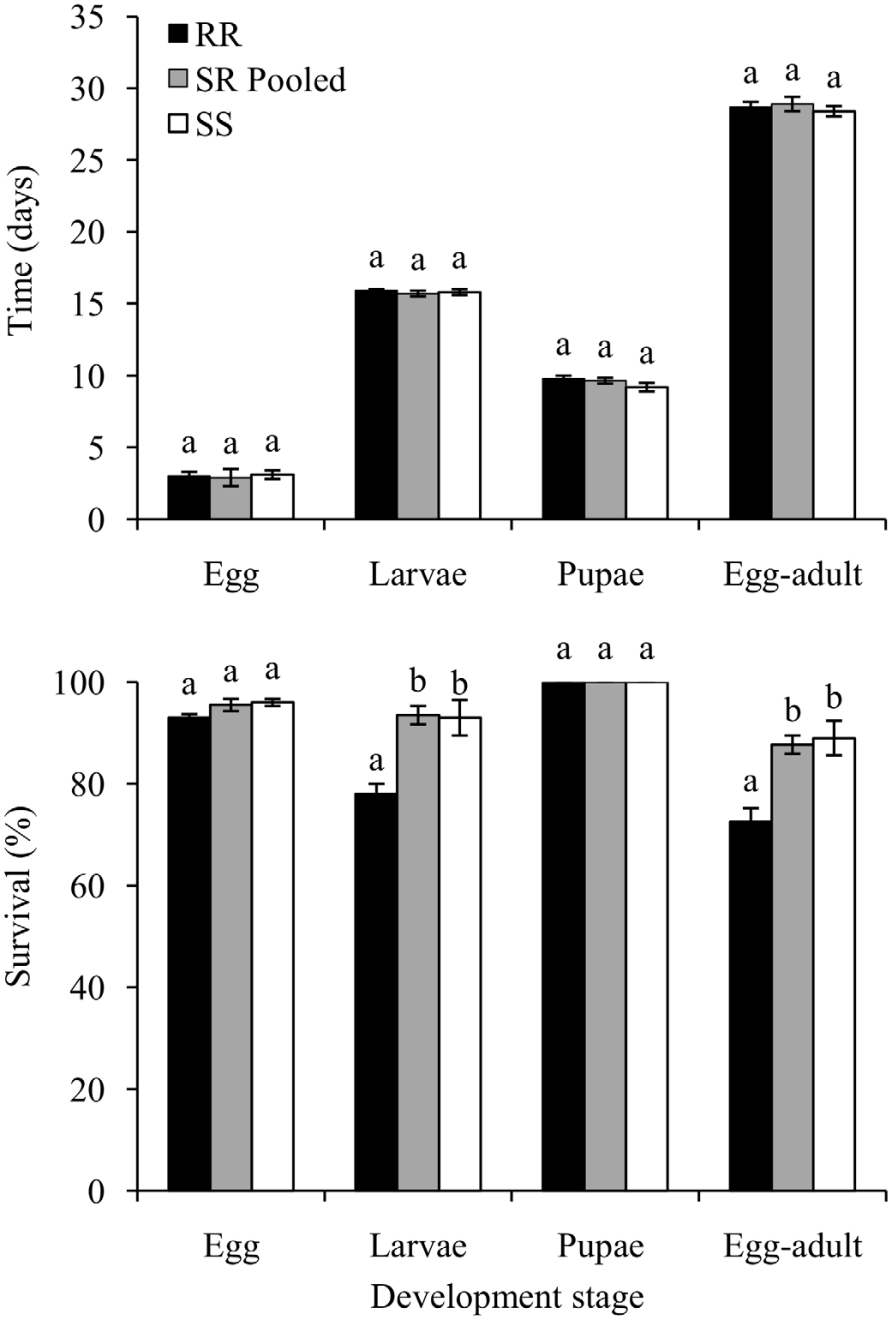
Comparison of fitness components of life stages of *S*. *frugiperda* strains reared on non-Bt corn. Within each development stage, bars with the same letter are not significantly different (LSD *t*-test; *P* > 0.05).

To investigate fitness costs associated with the resistance in the RR strain, life history traits were evaluated for the different *S*. *frugiperda* strains fed on non-Bt maize. There were no significant differences in the durations of the egg, larval and pupal periods and the duration from egg to adult (≈29 days) among the strains tested (SS, RR and SR Pooled; Fig 2; S6 Table), nor were differences observed in egg and pupal viability among the three strains. However, larval survivorship was about 15% lower for RR, which reduced the egg—adult viability relative to that of the other strains (<75% versus >87%) (Fig 2; S6 Table).

**Fig 3:**
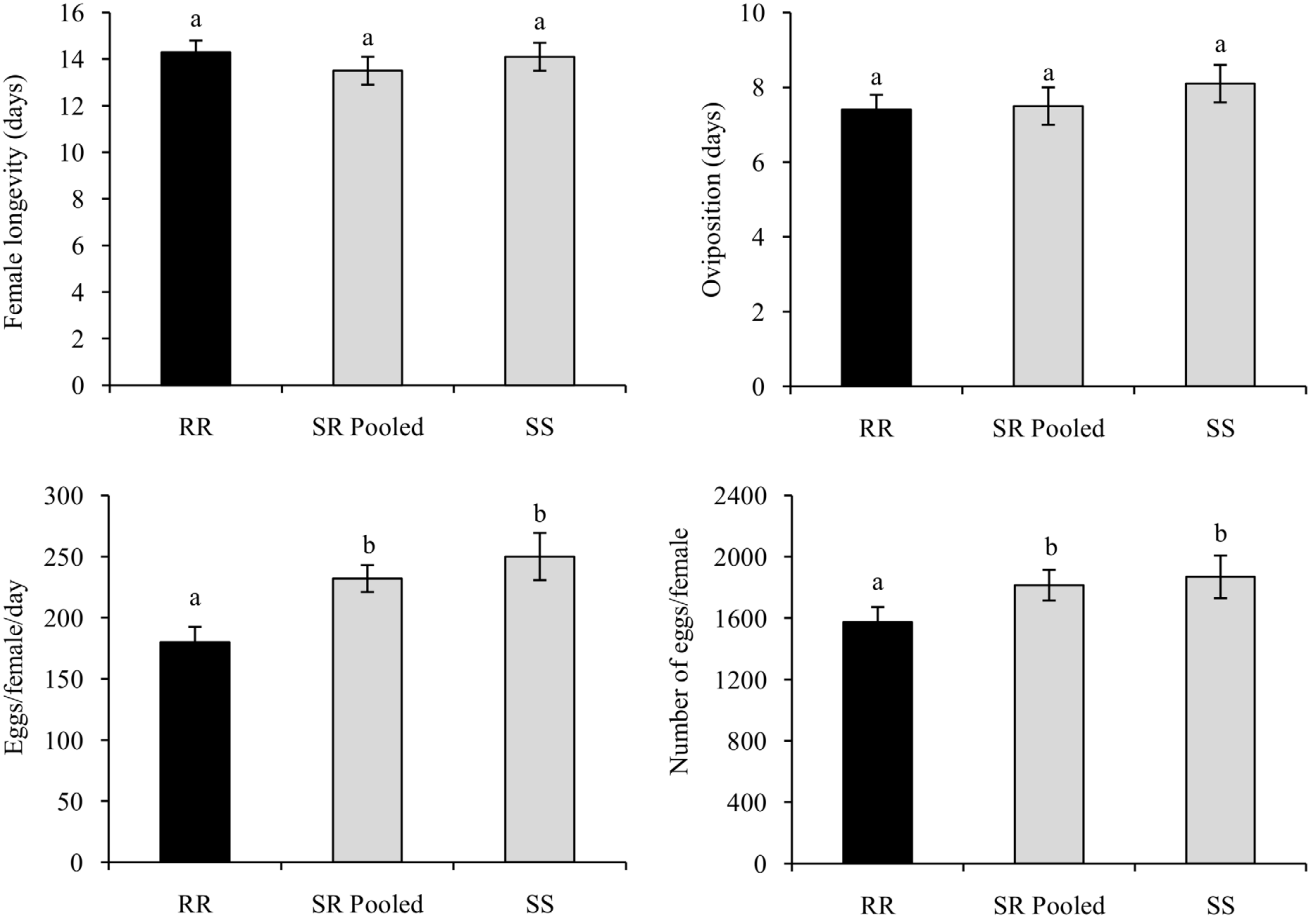
Comparison of fitness cost components of females of *S*. *frugiperda* strains reared on non-Bt corn. Bars with the same letter are not significantly different (LSD *t*-test; *P* > 0.05).

When fed on non-Bt maize, no significant differences were detected in female longevity (≈14 days) or oviposition period (8 days) (Fig 3; S7 Table). Also, there were no differences in sex ratio (female: male) among the strains (0.48–0.50). However, females of the resistant (RR) strain had decreased daily and total fecundity, which resulted in small reductions in reproductive rate when compared to the susceptible (SS) genotype and heterozygous strains (SR Pooled) (Fig 3; S7 Table).

The reduction in reproductive capacity affected some life table parameters (Table 6; S8 Table). The mean generation time (T) did not differ among the strains but the net reproductive rate (R_o_) was reduced by 31.7% in RR females relative to SS (Table 6; S8 Table). The SR Pooled strain had a similar net reproductive rate to SS (Table 6; S8 Table). Based on these findings, after ≈36 days of development (T), the SS and SR Pooled strains are expected to generate (R_o_ × λ) approximately 884 and 837 females from each female, respectively, while the RR strain would produce 599 females/female (Table 6; S8 Table). Therefore, the RR strain produced 32.2% and 28.4% less females from each female relative to the SS and RS (pooled) strains, respectively. The RR strain also had a significantly lower intrinsic rate of increase (r_m_), with 6% fewer females generated per female per day. Similarly, the finite rate of increase (λ) of the RR strain was significantly lower than that of the other strains (Table 6; S8 Table). Jakka et al. [45] demonstrated lack of fitness costs on a field-evolved *S*. *frugiperda* strain resistant to the Cry1F protein.

**Table 6:** Fertility life table parameters of *S*. *frugiperda* strains fed on non-Bt maize.

FAW strain	Fertility life table parameter^a^			
	T (days)	R_o_ (♀/♀)	r_m_ (♀/♀/day)	λ
RR	35.7 ± 0.2 a	503.4 ± 27.7 b	0.17 ± 0.001 b	1.19 ± 0.002 b
SR Pooled	36.6 ± 0.1 a	697.5 ± 34.8 a	0.18 ± 0.001 a	1.20 ± 0.001 a
SS	35.5 ± 0.4 a	736.7 ± 55.6 a	0.18 ± 0.002 a	1.20 ± 0.003 a

Values within a column followed by the same letter are not significantly different for two-tailed *t*-tests for pairwise group comparisons (*P* > 0.05).

^a^ T = mean generation time; R_o_ = net reproductive rate; r_m_ = intrinsic rate of increase and λ = finite rate of increase.

The genetic characterization of the resistance in the RR strain showed it to be autosomal with no sex linkage or maternal effects, and highly recessive. These results align with those of Storer et al. [28] and Farias et al. [29]. These authors characterized Cry1F resistance in *S*. *frugiperda* from Puerto Rico and Brazil, respectively. The RR strain described herein was originally sampled off Cry1F-expressing corn plants (field population BA27 in Table 1) and was shown to contain Cry1F resistance alleles [29]. After continuous selection of BA27 insects on MON 89034, the concentration-response assays (Table 3; Fig 1A and 1B) indicated significant differences between the SS and RR strains when exposed to Cry1A.105 but only small differences in susceptibility to Cry2Ab2 (Table 3; Fig 1C and 1D). The results also indicated that the Cry2Ab2 single-Bt corn event (Cry2A-P) was effective in controlling neonates of the RR strain (Table 5). Taken as a whole, the results indicate that the *S*. *frugiperda* population originally isolated from the field (BA27) had high-level Cry1F resistance. As shown in both the diet-incorporated bioassays and the leaf disc tests, the RR strain had increased survival on both Cry1Ab and Cry1A.105 relative to the SS strain, reflecting cross-resistance among these Cry1 proteins. Because Cry2Ab2 has a mode of action distinct from that of the Cry1 proteins [34], cross-resistance between Cry2Ab2 and Cry1F or Cry1A proteins is unlikely [38].

Consistent with the lack of significant resistance to Cry2Ab2, MON 89034 maize in combination with appropriate management practices continues to provide effective control of *S*. *frugiperda* in Brazil (unpublished data). These practices include scouting of *S*. *frugiperda* populations, planting of refuges and crop rotation (when applicable). Nevertheless, results presented herein indicate that current Cry1-based maize hybrids face a challenge in managing *S*. *frugiperda* in Brazil and highlight the importance of effective insect resistance management including the implementation and proper management of refuge areas.

## Materials and Methods

**Fig 4:**
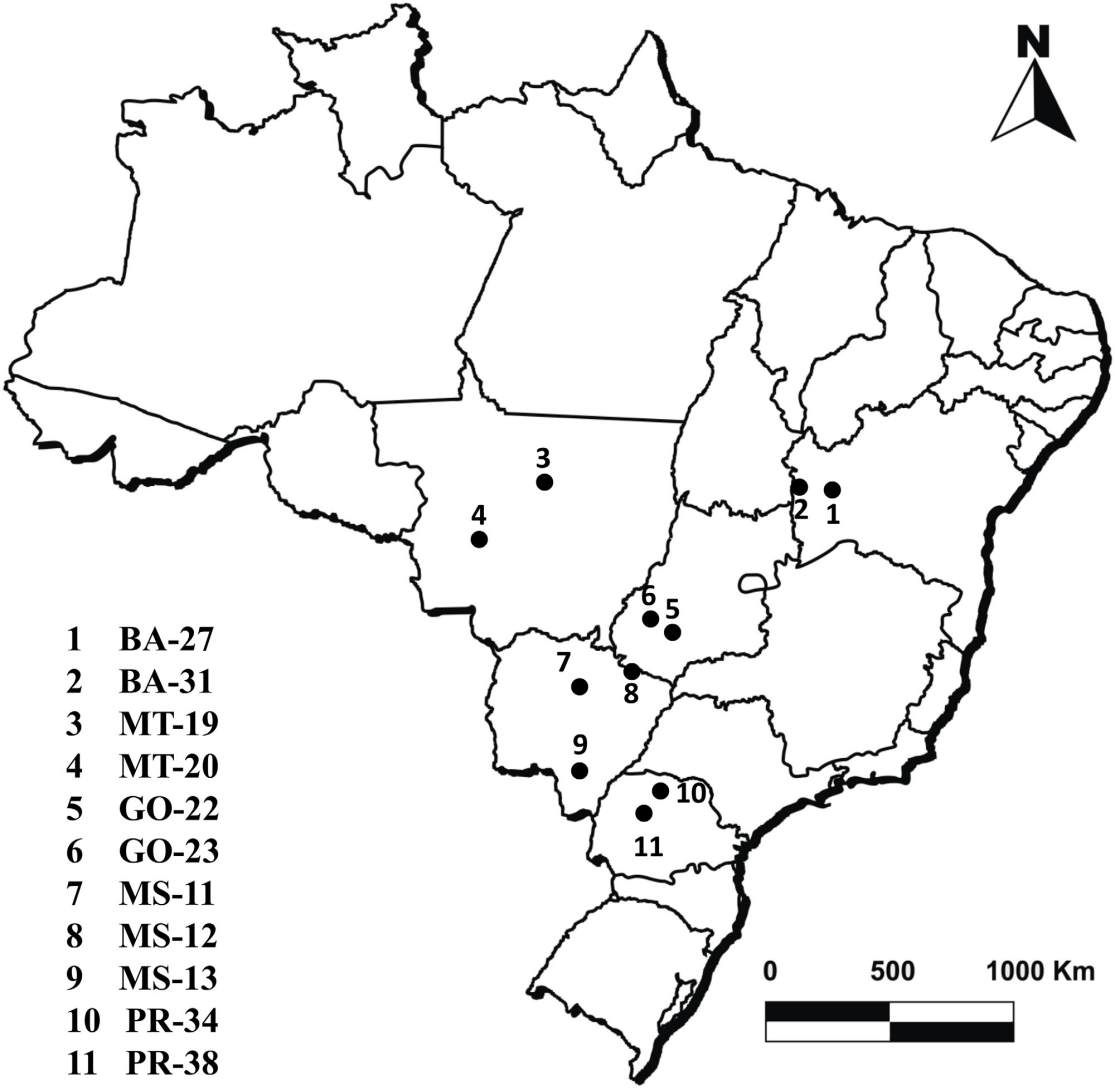
Distribution of populations of *Spodoptera frugiperda* used in F_2_ screen.

Permit access to collect material used in our research at various crop sites was granted by Sistema de Autorização e Informação em Biodiversidade (Sisbio) from the Brazilian Ministry of Environment to SGS Gravena (Sisbio License # 10018–1) and PROMIP (Sisbio License # 40380–2). Number of caterpillars collected and respective geographic coordinates of each location are listed in Fig 4 and Table 7.

**Table 7:** Geographic coordinates, total number of caterpillars sampled and date of sampling of field populations sampled for F_2_ screen.

Insect population	County/State	Farm	Latitude	Longitude	*n*	Date
BA27	São Desidério, BA	Grupo Mizote	13°00′78″S	46°09′83″W	480	Jan. 2012
BA31	Luís Eduardo Magalhães, BA	Circulo Verde	11°50′16″S	46°17′21″W	500	June 2012
GO22	Montividiu, GO	Ouro Verde	17°15′35″S	51°14′50″W	550	March 2012
GO23	Caiapônia, GO	Mata Alta	17°13′02″S	51°38′38″W	524	May 2012
MT19	Sinop, MT	_	11°51′32″S	55°35′30″W	568	April 2012
MT20	Campo Novo do Parecis, MT	Chapeco	13°24′47″S	57°57′51″W	570	April 2012
MS11	São Gabriel do Oeste, MS	_	19°23′37″S	54°33′49″W	486	March 2012
MS12	Chapadão do Sul, MS	Romulo Ideal	18°46′44″S	52°36′59″W	150	April 2012
MS13	Dourados, MS	Boa Vista	22°01′13″S	54°32′03″W	228	May 2012
PR34	Sabáudia, PR	Campo Bandeira	23°18′43″S	51°29′43″W	228	May 2012
PR38	Campo Mourão, PR	Grupo Integral	24°06′16″S	52°26′25″W	601	May 2012

### F_2_ screen

A total of 552 two-parent isofamilies of *S*. *frugiperda* were established from field collections in 2012 (Fig 4; Tables 1 and 7), which included 155 isofamilies from Bahia state (BA). Out of 155 BA families, 99 families (BA27) were sampled on Bt maize expressing Cry1F. F_2_ neonates of the families were screened on leaf tissues of MON 89034 maize plants excised from greenhouse-grown plants at V4–V8. Plants were grown under conditions conducive for maize development. A total of 120 neonates per family were tested. Bioassay trays containing maize leaf tissue and larvae of *S*. *frugiperda* were incubated in environmental chambers maintained at 27 ± 1°C, 60% RH and a 14h:10h (L:D) photoperiod. Every 2 days, fresh leaf tissue was added and larval survival recorded. (Iso)families were considered positive if any larvae completed their life cycle. Survivors of the positive (iso)families from the BA27 field population were kept under selection on MON 89034 leaves to establish the resistant (RR) strain.

Larvae of the RR strain were reared on MON 89034 corn leaves until the ninth generation, during which they were fed exclusively on MON 89034 corn leaves for the entire larval period. From the tenth generation onwards, larvae of the RR strain were fed until the third instar on MON 89034 leaves and then transferred to artificial diet [46] without Bt protein, where they remained until the pupal stage.

### Resistance characterization

Greenhouse trials were performed to evaluate the survival of susceptible (SS), resistant (RR) and heterozygous (S♂R♀ and S♀R♂) strains on MON 89034 and non-Bt maize. The expression of the Bt proteins on MON 89034 maize was confirmed using QuickStix for Cry1A and Cry2A in corn leaf & seed (Envirologix Inc., Portland, Maine, USA). The reference susceptible strain (SS) has been maintained in the laboratory for more than 10 years without selection pressure. Both of the heterozygous strains were obtained by reciprocal crosses between RR and SS strains. Plants in the greenhouse were cultivated in 4-L pots under conditions conducive for maize development. At the V4 stage, each plant was infested with one neonate larva (<24 h old). To prevent larval mobility, each plant was contained within a transparent plastic tube (1.0 m height × 0.30 m diameter) attached to the edge of each pot and covered at the top with a voile-type fabric. At 7 days after inoculation, larval survival was measured. Larvae that did not molt to the second instar were considered dead. Differences among strains were determined using least square means at α = 0.05 (PROC GLM [47]).To evaluate the inheritance of resistance, susceptible (SS), resistant (RR) and both heterozygous (S♂R♀ and S♀R♂) strains were used in diet-overlay bioassays with nine to ten concentrations of Cry1A.105 (0.15 to 16,000 ng/cm^2^ diet) or Cry2Ab2 (0.5 to 4,890 ng/cm^2^ diet). For the diet-overlay bioassay, concentration-mortality data were analyzed by Probit analysis to estimate the MIC_50_ (concentration that prevents 50% of larvae from molting to second instar) and the respective confidence interval (95% CI) using Polo Plus^®^ software [48]. Larval weight data were analyzed by nonlinear regression to estimate the EC_50_ (concentration that reduces larval biomass accumulation by 50%) and the respective confidence interval (95% CI) using JMP 9 Version 10 software [49]. MIC_50_ and EC_50_ values were considered significantly different among treatments when their 95% CI’s did not overlap. Resistance Ratios were calculated by dividing the MIC_50_ or EC_50_ of the RR, RS, SR or SR Pooled strain by the corresponding parameter for the SS strain.

The mortality data for RR, SS and reciprocal crosses between RR and SS (S♂R♀ and S♀R♂) strains were used to estimate the effective dominance (*D*_GIL_) of resistance to each protein using the method reported by Bourguet et al. [40]. Because the inheritance of resistance was autosomal, the SR Pooled strain was used for the calculation of *D*_GIL_. *D*_GIL_ values were calculated at 508.7 and 1,589.8 ng/cm^2^ of diet of Cry1A.105 and Cry2Ab2 protein, respectively. These concentrations were selected because they produced greater than 90% but less than 100% growth inhibition in the susceptible strain (SS) and lower response in the resistant strain (RR). Neonate larvae from each strain were tested individually in 128-well bioassays trays. Diet-overlay bioassays were performed as described above.

### Cross-resistance bioassays

Survival of the RR strain on other Bt products was examined with leaf disc bioassays using commercial products and experimental lines expressing single-Bt proteins: MON 810 (Cry1Ab), TC1507 (Cry1F), Cry1A.105 (Cry1A-P) and Cry2Ab2 (Cry2A-P). Both experimental lines (Cry1A-P and Cry2A-P) express levels of Bt proteins comparable to those in the MON 89034 event. Quality assurance of these Bt corn lines was maintained by PCR assay. All the corn lines tested were in the same relative maturity group, minimizing possible confounding effects and noise in the in planta bioassays. Completely expanded leaves were removed from the upper third of greenhouse-grown plants at the V5 stage. Leaf discs measuring 1.2 cm in diameter were cut using a metallic cutter and placed on a non-gelled mixture of water and agar at 2.5% (1 ml/well) in acrylic plates (Costar^®^) with 12 wells (Corning, Tewksbury, MA, USA). Leaf discs were separated from the water—agar layer by a filter paper disc. One neonate larva (<24 h old) was placed on each maize leaf disc using a fine brush. Plates were sealed with plastic film and placed in a climatic chamber (temperature: 27 ± 1°C; relative humidity: 60 ± 10%; photoperiod: 14h:10h light:dark). The experimental design was completely randomized with five replicates per treatment, consisting of 60 neonates tested for each maize event and *S*. *frugiperda* population. Larval mortality, weight reduction and growth inhibition relative to the control were recorded at 5 days after leaf disc infestation.

### Fitness cost

Susceptible (SS), resistant (RR) and heterozygous (SR) strains feeding on leaf tissues of non-Bt maize excised from greenhouse-grown maize plants at V6 were used to investigate the fitness costs of resistance. Methods for rearing larvae for this experiment followed what was described above. Fresh leaf tissue was added every 2 days. Each treatment consisted of 160 neonates (10 replicates of 16 larvae) from each strain. The following life history traits were evaluated: duration and survival rates of egg, larval and pupal periods; total cycle duration (egg to adult); female longevity; duration of oviposition; daily fecundity (eggs/female/day) and total fecundity (total eggs/female). All parameters and statistical procedures were analyzed as described by Bernardi et al. [50]. A life table was calculated by estimating the mean generation time (T), the net reproductive rate (R_o_), the intrinsic rate of increase (r_m_) and the finite rate of increase (λ). The life table parameters were estimated by the Jackknife method using SAS as described by Maia et al. [51].

## Supporting Information

S1 Table Number of two-parent families tested and positive lines of *S*. *frugiperda* (those with any offspring that survived on MON 89034 maize) identified using F2 screen method. (DOCX)

S2 Table Survival of *S*. *frugiperda* larvae per plant (mean ± SE) on MON 89034 maize and non-Bt near-isoline in greenhouse trials. (DOCX)

S3 Table Concentration-response and growth inhibition response (MIC50 and EC50; ng/cm2) of *S*. *frugiperda* in diet-overlay bioassays with purified Cry1A.105 and Cry2Ab2. (DOCX)

S4 Table Concentration-response of *S*. *frugiperda* in diet-overlay bioassays with purified Cry1A.105 and Cry2Ab2 proteins. (DOCX)

S5 Table Survival of *S*. *frugiperda* on leaf discs of different Bt and non-Bt maize plants. (DOCX)

S6 Table Comparison of fitness components of life stages of *S*. *frugiperda* strains reared on non-Bt corn. (DOCX)

S7 Table Comparison of fitness cost components of females of *S*. *frugiperda* strains reared on non-Bt corn. (DOCX)

S8 Table Fertility life table parameters of *S*. *frugiperda* strains fed on non-Bt maize. (DOCX)

## References

[1] Paterniani E. Sustainable agriculture in the tropics In: Rocha-Miranda CE, editor. Transition to global sustainability: the contribution of Brazilian science. Rio de Janeiro: Academia Brasileira de Ciências; 2000 pp. 181–194.

[2] Hill DS. Agricultural insect pests of the tropics and their control. 2nd ed. Cambridge University Press; 1983.

[3] Hollingsworth RG. Insect pest management of tropical versus temperate crops; patterns of similarities and differences in approach. Acta Hortic. 2011;894:45–56.

[4] Brookes G, Barfoot P. GM crops: global socio-economic and environmental impacts 1996–2012. Dorchester, UK: PG Economics; 2014 Available: www.pgeconomics.co.uk.

[5] James C. Global status of commercialized biotech/GM crops: 2014 ISAAA Brief No. 49. Ithaca, NY: ISAAA; 2014.

[6] Hutchison WD, Burkness EC, Mitchell PD, Moon RD, Leslie TW, Fleischer SJ, et al Areawide suppression of European corn borer with Bt maize reaps savings to non-Bt maize growers. Science 2010;330:222–225. 10.1126/science.1190242 20929774

[7] Naranjo SE. Effects of GM crops on non-target organisms In: Ricroch A, Chopra S, Fleischer SJ, editors. Plant biotechnology: experience and future prospects. Cham, Switzerland: Springer; 2014 pp. 129–142.

[8] Kathage J, Qaim M. Economic impacts and impact dynamics of Bt (*Bacillus thuringiensis*) cotton in India. Proc Natl Acad Sci USA. 2012;109(29):11652–11656. 10.1073/pnas.1203647109 22753493 PMC3406847

[9] Tabashnik BE, Brévault T, Carrière Y. Insect resistance to Bt crops: lessons from the first billion acres. Nat Biotechnol. 2013;31(6):510–521. 10.1038/nbt.2597 23752438

[10] Klümper W, Qaim M. A meta-analysis of the impacts of genetically modified crops. PLoS ONE 2014;9(11):e111629 10.1371/journal.pone.0111629 25365303 PMC4218791

[11] Lu Y, Wu K, Jiang Y, Guo Y, Desneux N. Widespread adoption of Bt cotton and insecticide decrease promotes biocontrol services. Nature 2012;487:362–365. 10.1038/nature11153 22722864

[12] Comas C, Lumbierres B, Pons X, Albajes R. No effects of *Bacillus thuringiensis* maize on nontarget organisms in the field in southern Europe: a meta-analysis of 26 arthropod taxa. Transgenic Res. 2014;23:135–143. 10.1007/s11248-013-9737-0 23904218

[13] Lewinsohn TM, Prado PI. How many species are there in Brazil? Conserv Biol. 2005;19:619–624.

[14] FAO. 2015. Available: http://www.fao.org/home/en/. Accessed 3 May 2015.

[15] Pereira PAA, Martha GB Jr, Santana CAM, Alves E. The development of Brazilian agriculture: future technological challenges and opportunities. Agriculture & Food Security 2012;1:4.

[16] CTNBio, Comissão Técnica Nacional de Biossegurança. Commercial release of genetically modified corn, Guardian corn (MON 810). In: Technical opinion n° 1.100/2007; 2007. Available: http://www.ctnbio.gov.br/index.php/content/view/11763.html. Accessed 27 March 2015.

[17] Céleres. Informativo Biotecnologia, IB14.03, 16 12 2014 Uberlândia, MG, Brazil: Céleres; 2014.

[18] Cruz I, Figueiredo MLC, Silva RB, Silva IF, Paula CS, Foster JE. Using sex pheromone traps in the decision-making process for pesticide application against fall armyworm (*Spodoptera frugiperda* [Smith] [Lepidoptera: Noctuidae]) larvae in maize. International Journal of Pest Management 2012;58(1):83–90.

[19] Diez-Rodriguez GI, Omoto C. Inheritance of lambda-cyhalothrin resistance in *Spodoptera frugiperda* (J.E. Smith) (Lepidoptera: Noctuidae). Neotrop Entomol. 2001;30(2):311–316.

[20] Carvalho RA, Omoto C, Field LM, Williamson MS, Bass C. Investigating the molecular mechanisms of organophosphate and pyrethroid resistance in the fall armyworm *Spodoptera frugiperda* . PLoS ONE 2013;8(4):e62268 10.1371/journal.pone.0062268 23614047 PMC3629120

[21] Mendes SM, Waquil JM, Marucci RC, Boregas KGB. Avaliação da incidência de organismos alvo e não alvo em milho *Bt* (Cry 1Ab) em condições de campo em Sete Lagoas-MG Circular Técnica 128. Sete Lagoas, MG, Brazil: Embrapa; 2009.

[22] Okumura RS, Mariano DC, Dallacort R, Zorzenoni TO, Zaccheo PVC, Neto CFO, et al Agronomic efficiency of *Bacillus thuringiensis* (Bt) maize hybrids in pests control on Lucas do Rio Verde city, State of Mato Grosso, Brazil. Afr J Agric Res. 2013;8(19):2232–2239.

[23] Waquil JM, Dourado PM, Carvalho RA, Oliveira WS, Berger GU, Head GP, Martinelli S. Manejo de lepidópteros-praga na cultura do milho com o evento Bt piramidado Cry1A.105 e Cry2Ab2. Pesquisa Agropecuária Brasileira. 2013;48(12):1529–1537.

[24] Carpenter JE. Peer-reviewed surveys indicate positive impact of commercialized GM crops. Nat Biotechnol. 2010;28(4):319–321. 10.1038/nbt0410-319 20379171

[25] McGaughey WH, Whalon ME. Managing insect resistance to *Bacillus thuringiensis* toxins. Science. 1992;258:1451–1455. 17755107 10.1126/science.258.5087.1451

[26] Gould F. Sustainability of transgenic insecticidal cultivars: integrating pest genetics and ecology. Annu Rev Entomol. 1998;43:701–726. 15012402 10.1146/annurev.ento.43.1.701

[27] Van Rensburg JBJ. First report of field resistance by the stem borer, *Busseola fusca* (Fuller) to Bt-transgenic maize. S Afr J Plant Soil. 2007;24:147–151.

[28] Storer NP, Babcock JM, Schlenz M, Meade T, Thompson GD, Bing JW, Huckaba RM. Discovery and characterization of field resistance to *Bt* maize: *Spodoptera frugiperda* (Lepidoptera: Noctuidae) in Puerto Rico. J Econ Entomol. 2010;103:1031–1038. 20857709 10.1603/ec10040

[29] Farias JR, Andow DA, Horikoshi RJ, Sorgatto RJ, Fresia P, Santos AC, Omoto C. Field-evolved resistance to Cry1F maize by *Spodoptera frugiperda* (Lepidoptera: Noctuidae) in Brazil. Crop Prot. 2014;64:150–158.

[30] Dhurua S, Gujar GT. Field-evolved resistance to *Bt* toxin Cry1Ac in the pink bollworm, *Pectinophora gossypiella* (Saunders) (Lepidoptera: Gelechiidae), from India. Pest Manag Sci. 2011;67:898–903. 10.1002/ps.2127 21438121

[31] Gassmann AJ, Petzold-Maxwell JL, Keweshan RS, Dunbar MW. Field-evolved resistance to Bt maize by western corn rootworm, PLoS ONE. 2011;6(7):e22629 10.1371/journal.pone.0022629 21829470 PMC3146474

[32] Martinelli S, Omoto C. Resistência de insetos a plantas geneticamente modificadas. Biotecnolgia Ciência e Desenvolvimento. 2005;34:67–77.

[33] Farias JR, Horikoshi RJ, Santos AC, Omoto C. Geographical and temporal variability in susceptibility to Cry1F toxin from *Bacillus thuringiensis* in *Spodoptera frugiperda* (Lepidoptera: Noctuidae) populations in Brazil. J Econ Entomol. 2014;107(6):2182–2189. 26470084 10.1603/EC14190

[34] Hernández-Rodríguez CS, Hernández-Martínez P, Van Rie J, Escriche B, Ferré J. Shared midgut binding sites for Cry1A.105, Cry1Aa, Cry1Ab, Cry1Ac and Cry1Fa proteins from *Bacillus thuringiensis* in two important corn pests, *Ostrinia nubilalis* and *Spodoptera frugiperda* . PLoS ONE. 2013;8(7):e68164 10.1371/journal.pone.0068164 23861865 PMC3702569

[35] Niu Y, Meagher RL Jr, Yang F, Huang F. Susceptibility of field populations of the fall armyworm (Lepidoptera: Noctuidae) from Florida and Puerto Rico to purified Cry1F protein and corn leaf tissue containing single and pyramided Bt genes. Fla Entomol. 2013;96(3):701–713.

[36] Yang F, Qureshi JA, Leonard BR, Head GP, Niu Y, Huang F. Susceptibility of Louisiana and Florida populations of *Spodoptera frugiperda* (Lepidoptera: Noctuidae) to pyramided Bt corn containing Genuity^®^VT Double Pro^™^ and SmartStax^™^ traits. Fla Entomol. 2013;96(3):714–723.

[37] Huang F, Qureshi JA, Meagher RL Jr, Reisig DD, Head GP, Andow DA, et al Cry1F resistance in fall armyworm *Spodoptera frugiperda*: single gene versus pyramided Bt maize. PLoS ONE. 2014;9(11):e112958 10.1371/journal.pone.0112958 25401494 PMC4234506

[38] Monnerat R, Martins E, Macedo C, Queiroz P, Praça L, Soares CM, et al Evidence of field-evolved resistance of *Spodoptera frugiperda* to Bt corn expressing Cry1F in Brazil that is still sensitive to modified Bt toxins. PLoS ONE. 2015;10(4):e0119544 10.1371/journal.pone.0119544 25830928 PMC4382162

[39] Andow DA, Alstad DN. F_2_ screen for rare resistance alleles. J Econ Entomol. 1998;91:572–578.

[40] Bourguet D, Genissel A, Raymond M. Insecticide resistance and dominance levels. J Econ Entomol. 2000;93:1588–1595. 11142285 10.1603/0022-0493-93.6.1588

[41] Biosafety Clearing-House. Gene and DNA sequence, Cry1A.105; 2015 [last update 2014-02-24]. Available: https://bch.cbd.int/database/record.shtml?documentid=43771. Accessed 29 May 2015.

[42] Storer NP, Thompson GD, Head GP. Application of pyramided traits against Lepidoptera in insect resistance management for Bt crops. GM Crops Food. 2012;3(3):154–162. 10.4161/gmcr.20945 22688687

[43] Vélez AM, Spencer TA, Alves AP, Moellenbeck D, Meagher RL, Chirakkal H, Siegfried BD. Inheritance of Cry1F resistance, cross-resistance and frequency of resistant alleles in *Spodoptera frugiperda* (Lepidoptera: Noctuidae). Bull Entomol Res. 2013;103:700–713. 10.1017/S0007485313000448 23941623

[44] Niu Y, Yang F, Dangal V, Huang F. Larval survival and plant injury of Cry1F-susceptible, -resistant, and -heterozygous fall armyworm (Lepidoptera: Noctuidae) on non-Bt and Bt corn containing single or pyramided genes. Crop Prot. 2014;59:22–28.

[45] Jakka SRK, Knight VR, Jurat-Fuentes JL. Fitness costs associated with field-evolved resistance to Bt maize in *Spodoptera frugiperda* (Lepidoptera: Noctuidae). Journal of Economic Entomology 2014;107, 342–351. 24665719 10.1603/ec13326

[46] Kasten JRP, Precetti AACM, Parra JRP. Dados biológicos comparativos de *Spodoptera frugiperda* (J.E. Smith, 1797) em duas dietas artificiais e substrato natural. Rev. Agric. 1978;53: 68–78.

[47] SAS Institute. Statistical analysis system: getting started with the SAS learning. Cary, NC: SAS Institute; 2000.

[48] Robertson JL, Preisler HK. Pesticide bioassays with arthropods. 1st ed. London: CRC Press; 1992.

[49] SAS Institute. JMP Software—Introductory guide version 9.0. Cary, NC: SAS Institute; 2010.

[50] Bernardi O, Sorgatto RJ, Barbosa AD, Domingues FA, Dourado PM, Carvalho RA, et al Low susceptibility of *Spodoptera cosmioides*, *Spodoptera eridania* and *Spodoptera frugiperda* (Lepidoptera: Noctuidae) to genetically-modified soybean expressing Cry1Ac protein. Crop Prot. 2014;58:33–40.

[51] Maia AHN, Luiz AJB, Campanhola C. Statistical inference on associated fertility life table parameters using jackknife technique: computational aspects. J Econ Entomol. 2000;93(2):511–518. 10826207 10.1603/0022-0493-93.2.511

