# Supplementary material for: Cross-Resistance between Cry1 Proteins in Fall Armyworm (Spodoptera frugiperda) May Affect the Durability of Current Pyramided Bt Maize Hybrids in Brazil
Source: PLoS One. 2015 Oct 16;10(10):e0140130. doi: 10.1371/journal.pone.0140130 (PMC4608726; doi:10.1371/journal.pone.0140130)
Supplement: S1 Table — (DOCX) [file pone.0140130.s001.docx]

**S1 Table.** Number of two-parent families tested and positive lines of *S. frugiperda* (those with any offspring that survived on MON 89034 maize) identified using F_2_ screen method.

| Number isofamilies tested | Insect population | Neonates per  family | Number of adults | Positive  F_2_ lines |
| --- | --- | --- | --- | --- |
| 1 | BAH 27 | 120 | 0 | 0 |
| 2 | BAH 27 | 120 | 2 | 1 |
| 3 | BAH 27 | 120 | 3 | 1 |
| 4 | BAH 27 | 120 | 1 | 1 |
| 5 | BAH 27 | 120 | 1 | 1 |
| 6 | BAH 27 | 120 | 1 | 1 |
| 7 | BAH 27 | 120 | 6 | 1 |
| 8 | BAH 27 | 120 | 0 | 0 |
| 9 | BAH 27 | 120 | 1 | 1 |
| 10 | BAH 27 | 120 | 0 | 0 |
| 11 | BAH 27 | 120 | 1 | 1 |
| 12 | BAH 27 | 120 | 0 | 0 |
| 13 | BAH 27 | 120 | 1 | 1 |
| 14 | BAH 27 | 120 | 0 | 0 |
| 15 | BAH 27 | 120 | 3 | 1 |
| 16 | BAH 27 | 120 | 3 | 1 |
| 17 | BAH 27 | 120 | 1 | 1 |
| 18 | BAH 27 | 120 | 0 | 0 |
| 19 | BAH 27 | 120 | 4 | 1 |
| 20 | BAH 27 | 120 | 0 | 0 |
| 21 | BAH 27 | 120 | 0 | 0 |
| 22 | BAH 27 | 120 | 7 | 1 |
| 23 | BAH 27 | 120 | 0 | 0 |
| 24 | BAH 27 | 120 | 0 | 0 |
| 25 | BAH 27 | 120 | 4 | 1 |
| 26 | BAH 27 | 120 | 0 | 0 |
| 27 | BAH 27 | 120 | 1 | 1 |
| 28 | BAH 27 | 120 | 2 | 1 |
| 29 | BAH 27 | 60 | 0 | 0 |
| 30 | BAH 27 | 120 | 3 | 1 |
| 31 | BAH 27 | 120 | 0 | 0 |
| 32 | BAH 27 | 120 | 1 | 1 |
| 33 | BAH 27 | 120 | 1 | 1 |
| 34 | BAH 27 | 120 | 0 | 0 |
| 35 | BAH 27 | 120 | 1 | 1 |
| 36 | BAH 27 | 120 | 1 | 1 |
| 37 | BAH 27 | 120 | 2 | 1 |
| 38 | BAH 27 | 120 | 0 | 0 |
| 39 | BAH 27 | 120 | 6 | 1 |
| 40 | BAH 27 | 120 | 0 | 0 |
| 41 | BAH 27 | 120 | 0 | 0 |
| 42 | BAH 27 | 120 | 2 | 1 |
| 43 | BAH 27 | 120 | 0 | 0 |
| 44 | BAH 27 | 120 | 4 | 1 |
| 45 | BAH 27 | 120 | 2 | 1 |
| 46 | BAH 27 | 120 | 0 | 0 |
| 47 | BAH 27 | 120 | 0 | 0 |
| 48 | BAH 27 | 120 | 0 | 0 |
| 49 | BAH 27 | 120 | 0 | 0 |
| 50 | BAH 27 | 120 | 0 | 0 |
| 51 | BAH 27 | 120 | 0 | 0 |
| 52 | BAH 27 | 120 | 0 | 0 |
| 53 | BAH 27 | 120 | 6 | 1 |
| 54 | BAH 27 | 120 | 8 | 1 |
| 55 | BAH 27 | 120 | 2 | 1 |
| 56 | BAH 27 | 120 | 0 | 0 |
| 57 | BAH 27 | 120 | 0 | 0 |
| 58 | BAH 27 | 120 | 0 | 0 |
| 59 | BAH 27 | 120 | 0 | 0 |
| 60 | BAH 27 | 120 | 0 | 0 |
| 61 | BAH 27 | 120 | 0 | 0 |
| 62 | BAH 27 | 120 | 0 | 0 |
| 63 | BAH 27 | 120 | 1 | 1 |
| 64 | BAH 27 | 120 | 1 | 1 |
| 65 | BAH 27 | 120 | 0 | 0 |
| 66 | BAH 27 | 120 | 0 | 0 |
| 67 | BAH 27 | 120 | 1 | 1 |
| 68 | BAH 27 | 120 | 0 | 0 |
| 69 | BAH 27 | 120 | 1 | 1 |
| 70 | BAH 27 | 120 | 0 | 0 |
| 71 | BAH 27 | 120 | 0 | 0 |
| 72 | BAH 27 | 120 | 4 | 1 |
| 73 | BAH 27 | 120 | 0 | 0 |
| 74 | BAH 27 | 120 | 0 | 0 |
| 75 | BAH 27 | 120 | 0 | 0 |
| 76 | BAH 27 | 120 | 0 | 0 |
| 77 | BAH 27 | 120 | 3 | 1 |
| 78 | BAH 27 | 120 | 0 | 0 |
| 79 | BAH 27 | 120 | 0 | 0 |
| 80 | BAH 27 | 120 | 1 | 1 |
| 81 | BAH 27 | 120 | 0 | 0 |
| 82 | BAH 27 | 120 | 1 | 1 |
| 83 | BAH 27 | 120 | 1 | 1 |
| 84 | BAH 27 | 120 | 0 | 0 |
| 85 | BAH 27 | 120 | 0 | 0 |
| 86 | BAH 27 | 120 | 0 | 0 |
| 87 | BAH 27 | 120 | 0 | 0 |
| 88 | BAH 27 | 120 | 1 | 1 |
| 89 | BAH 27 | 120 | 0 | 0 |
| 90 | BAH 27 | 120 | 0 | 0 |
| 91 | BAH 27 | 120 | 0 | 0 |
| 92 | BAH 27 | 120 | 0 | 0 |
| 93 | BAH 27 | 120 | 0 | 0 |
| 94 | BAH 27 | 120 | 0 | 0 |
| 95 | BAH 27 | 120 | 0 | 0 |
| 96 | BAH 27 | 120 | 0 | 0 |
| 97 | BAH 27 | 120 | 0 | 0 |
| 98 | BAH 27 | 120 | 0 | 0 |
| 99 | BAH 27 | 120 | 1 | 1 |
|  |  | | | |
| 1 | BAH 31 | 120 | 0 | 0 |
| 2 | BAH 31 | 120 | 0 | 0 |
| 3 | BAH 31 | 120 | 0 | 0 |
| 4 | BAH 31 | 120 | 1 | 1 |
| 5 | BAH 31 | 120 | 0 | 0 |
| 6 | BAH 31 | 120 | 0 | 0 |
| 7 | BAH 31 | 120 | 0 | 0 |
| 8 | BAH 31 | 120 | 1 | 1 |
| 9 | BAH 31 | 120 | 0 | 0 |
| 10 | BAH 31 | 120 | 0 | 0 |
| 11 | BAH 31 | 120 | 0 | 0 |
| 12 | BAH 31 | 120 | 0 | 0 |
| 13 | BAH 31 | 120 | 1 | 1 |
| 14 | BAH 31 | 120 | 0 | 0 |
| 15 | BAH 31 | 120 | 1 | 1 |
| 16 | BAH 31 | 120 | 0 | 0 |
| 17 | BAH 31 | 120 | 1 | 1 |
| 18 | BAH 31 | 120 | 0 | 0 |
| 19 | BAH 31 | 120 | 0 | 0 |
| 20 | BAH 31 | 120 | 1 | 1 |
| 21 | BAH 31 | 120 | 0 | 0 |
| 22 | BAH 31 | 120 | 0 | 0 |
| 23 | BAH 31 | 120 | 0 | 0 |
| 24 | BAH 31 | 120 | 1 | 1 |
| 25 | BAH 31 | 120 | 0 | 0 |
| 26 | BAH 31 | 120 | 1 | 1 |
| 27 | BAH 31 | 120 | 0 | 0 |
| 28 | BAH 31 | 120 | 0 | 0 |
| 29 | BAH 31 | 120 | 0 | 0 |
| 30 | BAH 31 | 120 | 0 | 0 |
| 31 | BAH 31 | 120 | 1 | 1 |
| 32 | BAH 31 | 120 | 1 | 1 |
| 33 | BAH 31 | 120 | 0 | 0 |
| 34 | BAH 31 | 120 | 0 | 0 |
| 35 | BAH 31 | 120 | 0 | 0 |
| 36 | BAH 31 | 120 | 0 | 0 |
| 37 | BAH 31 | 120 | 0 | 0 |
| 38 | BAH 31 | 120 | 1 | 1 |
| 39 | BAH 31 | 120 | 0 | 0 |
| 40 | BAH 31 | 120 | 0 | 0 |
| 41 | BAH 31 | 120 | 1 | 1 |
| 42 | BAH 31 | 120 | 0 | 0 |
| 43 | BAH 31 | 120 | 0 | 0 |
| 44 | BAH 31 | 120 | 0 | 0 |
| 45 | BAH 31 | 120 | 0 | 0 |
| 46 | BAH 31 | 120 | 0 | 0 |
| 47 | BAH 31 | 120 | 2 | 1 |
| 48 | BAH 31 | 120 | 1 | 1 |
| 49 | BAH 31 | 120 | 0 | 0 |
| 50 | BAH 31 | 120 | 0 | 0 |
| 51 | BAH 31 | 120 | 0 | 0 |
| 52 | BAH 31 | 120 | 1 | 1 |
| 53 | BAH 31 | 120 | 2 | 1 |
| 54 | BAH 31 | 120 | 1 | 1 |
| 55 | BAH 31 | 120 | 0 | 0 |
| 56 | BAH 31 | 120 | 0 | 0 |
|  |  | | | |
| 1 | G0 22 | 120 | 0 | 0 |
| 2 | G0 22 | 120 | 0 | 0 |
| 3 | G0 22 | 120 | 0 | 0 |
| 4 | G0 22 | 120 | 0 | 0 |
| 5 | G0 22 | 120 | 0 | 0 |
| 6 | G0 22 | 120 | 0 | 0 |
| 7 | G0 22 | 120 | 0 | 0 |
| 8 | G0 22 | 120 | 0 | 0 |
| 9 | G0 22 | 120 | 0 | 0 |
| 10 | G0 22 | 120 | 0 | 0 |
| 11 | G0 22 | 120 | 0 | 0 |
| 12 | G0 22 | 120 | 0 | 0 |
| 13 | G0 22 | 120 | 0 | 0 |
| 14 | G0 22 | 120 | 0 | 0 |
| 15 | G0 22 | 120 | 5 | 1 |
| 16 | G0 22 | 120 | 0 | 0 |
| 17 | G0 22 | 120 | 0 | 0 |
| 18 | G0 22 | 120 | 0 | 0 |
| 19 | G0 22 | 120 | 0 | 0 |
| 20 | G0 22 | 120 | 0 | 0 |
| 21 | G0 22 | 120 | 0 | 0 |
| 22 | G0 22 | 120 | 2 | 1 |
| 23 | G0 22 | 120 | 0 | 0 |
| 24 | G0 22 | 120 | 0 | 0 |
| 25 | G0 22 | 120 | 0 | 0 |
| 26 | G0 22 | 120 | 0 | 0 |
| 27 | G0 22 | 120 | 0 | 0 |
| 28 | G0 22 | 120 | 0 | 0 |
| 29 | G0 22 | 120 | 1 | 1 |
| 30 | G0 22 | 120 | 1 | 1 |
| 31 | G0 22 | 120 | 0 | 0 |
| 32 | G0 22 | 120 | 0 | 0 |
| 33 | G0 22 | 120 | 0 | 0 |
| 34 | G0 22 | 120 | 0 | 0 |
| 35 | G0 22 | 120 | 0 | 0 |
| 36 | G0 22 | 120 | 5 | 1 |
| 37 | G0 22 | 120 | 0 | 0 |
| 38 | G0 22 | 120 | 0 | 0 |
| 39 | G0 22 | 120 | 0 | 0 |
| 40 | G0 22 | 120 | 0 | 0 |
| 41 | G0 22 | 120 | 0 | 0 |
| 42 | G0 22 | 120 | 0 | 0 |
| 43 | G0 22 | 120 | 0 | 0 |
| 44 | G0 22 | 120 | 0 | 0 |
| 45 | G0 22 | 120 | 0 | 0 |
| 46 | G0 22 | 120 | 0 | 0 |
| 47 | G0 22 | 120 | 2 | 1 |
| 48 | G0 22 | 120 | 0 | 0 |
| 49 | G0 22 | 120 | 0 | 0 |
| 50 | G0 22 | 120 | 0 | 0 |
| 51 | G0 22 | 120 | 0 | 0 |
| 52 | G0 22 | 120 | 0 | 0 |
| 53 | G0 22 | 120 | 0 | 0 |
| 54 | G0 22 | 120 | 0 | 0 |
| 55 | G0 22 | 120 | 1 | 1 |
| 56 | G0 22 | 120 | 0 | 0 |
| 57 | G0 22 | 120 | 0 | 0 |
| 58 | G0 22 | 120 | 0 | 0 |
| 59 | G0 22 | 120 | 0 | 0 |
| 60 | G0 22 | 120 | 0 | 0 |
| 61 | G0 22 | 120 | 0 | 0 |
| 62 | G0 22 | 120 | 0 | 0 |
| 63 | G0 22 | 120 | 0 | 0 |
| 64 | G0 22 | 120 | 0 | 0 |
| 65 | G0 22 | 120 | 0 | 0 |
| 66 | G0 22 | 120 | 0 | 0 |
| 67 | G0 22 | 120 | 0 | 0 |
| 68 | G0 22 | 120 | 0 | 0 |
| 69 | G0 22 | 120 | 0 | 0 |
| 70 | G0 22 | 120 | 0 | 0 |
| 71 | G0 22 | 120 | 0 | 0 |
| 72 | G0 22 | 120 | 0 | 0 |
| 73 | G0 22 | 120 | 0 | 0 |
| 74 | G0 22 | 120 | 0 | 0 |
|  |  | | | |
| 1 | G0 23 | 120 | 0 | 0 |
| 2 | G0 23 | 120 | 0 | 0 |
| 3 | G0 23 | 120 | 0 | 0 |
| 4 | G0 23 | 120 | 0 | 0 |
| 5 | G0 23 | 120 | 2 | 1 |
| 6 | G0 23 | 120 | 0 | 0 |
| 7 | G0 23 | 60 | 0 | 0 |
| 8 | G0 23 | 120 | 0 | 0 |
| 9 | G0 23 | 120 | 0 | 0 |
| 10 | G0 23 | 120 | 0 | 0 |
| 11 | G0 23 | 80 | 0 | 0 |
| 12 | G0 23 | 120 | 0 | 0 |
| 13 | G0 23 | 120 | 0 | 0 |
| 14 | G0 23 | 120 | 0 | 0 |
| 15 | G0 23 | 120 | 0 | 0 |
| 16 | G0 23 | 120 | 0 | 0 |
| 17 | G0 23 | 120 | 0 | 0 |
| 18 | G0 23 | 120 | 0 | 0 |
| 19 | G0 23 | 120 | 0 | 0 |
| 20 | G0 23 | 120 | 0 | 0 |
| 21 | G0 23 | 120 | 0 | 0 |
| 22 | G0 23 | 120 | 0 | 0 |
| 23 | G0 23 | 120 | 0 | 0 |
| 24 | G0 23 | 120 | 0 | 0 |
| 25 | G0 23 | 120 | 0 | 0 |
| 26 | G0 23 | 120 | 0 | 0 |
| 27 | G0 23 | 120 | 0 | 0 |
| 28 | G0 23 | 120 | 0 | 0 |
| 29 | G0 23 | 120 | 0 | 0 |
| 30 | G0 23 | 120 | 0 | 0 |
| 31 | G0 23 | 120 | 0 | 0 |
| 32 | G0 23 | 120 | 0 | 0 |
| 33 | G0 23 | 120 | 0 | 0 |
| 34 | G0 23 | 120 | 0 | 0 |
| 35 | G0 23 | 120 | 0 | 0 |
| 36 | G0 23 | 120 | 0 | 0 |
| 37 | G0 23 | 120 | 0 | 0 |
| 38 | G0 23 | 120 | 0 | 0 |
| 39 | G0 23 | 120 | 0 | 0 |
| 40 | G0 23 | 120 | 0 | 0 |
| 41 | G0 23 | 120 | 0 | 0 |
| 42 | G0 23 | 120 | 0 | 0 |
| 43 | G0 23 | 120 | 0 | 0 |
| 44 | G0 23 | 120 | 0 | 0 |
| 45 | G0 23 | 120 | 0 | 0 |
| 46 | G0 23 | 120 | 0 | 0 |
| 47 | G0 23 | 120 | 0 | 0 |
| 48 | G0 23 | 120 | 0 | 0 |
| 49 | G0 23 | 120 | 0 | 0 |
| 50 | G0 23 | 120 | 0 | 0 |
| 51 | G0 23 | 120 | 0 | 0 |
| 52 | G0 23 | 120 | 0 | 0 |
| 53 | G0 23 | 120 | 0 | 0 |
| 54 | G0 23 | 120 | 0 | 0 |
| 55 | G0 23 | 120 | 0 | 0 |
| 56 | G0 23 | 120 | 0 | 0 |
|  |  | | | |
| 1 | MT 19 | 120 | 0 | 0 |
| 2 | MT 19 | 120 | 0 | 0 |
| 3 | MT 19 | 120 | 0 | 0 |
| 4 | MT 19 | 120 | 0 | 0 |
| 5 | MT 19 | 120 | 0 | 0 |
| 6 | MT 19 | 120 | 0 | 0 |
| 7 | MT 19 | 120 | 0 | 0 |
| 8 | MT 19 | 120 | 0 | 0 |
| 9 | MT 19 | 120 | 0 | 0 |
| 10 | MT 19 | 120 | 0 | 0 |
| 11 | MT 19 | 120 | 0 | 0 |
| 12 | MT 19 | 120 | 0 | 0 |
| 13 | MT 19 | 120 | 0 | 0 |
| 14 | MT 19 | 120 | 0 | 0 |
| 15 | MT 19 | 120 | 0 | 0 |
| 16 | MT 19 | 120 | 0 | 0 |
| 17 | MT 19 | 120 | 0 | 0 |
| 18 | MT 19 | 120 | 0 | 0 |
| 19 | MT 19 | 120 | 0 | 0 |
| 20 | MT 19 | 120 | 0 | 0 |
| 21 | MT 19 | 120 | 0 | 0 |
| 22 | MT 19 | 120 | 0 | 0 |
| 23 | MT 19 | 120 | 0 | 0 |
| 24 | MT 19 | 120 | 0 | 0 |
| 25 | MT 19 | 120 | 0 | 0 |
| 26 | MT 19 | 120 | 0 | 0 |
|  |  |  |  |  |
| 1 | MT 20 | 120 | 0 | 0 |
| 2 | MT 20 | 120 | 0 | 0 |
| 3 | MT 20 | 120 | 0 | 0 |
| 4 | MT 20 | 120 | 0 | 0 |
| 5 | MT 20 | 60 | 0 | 0 |
| 6 | MT 20 | 120 | 0 | 0 |
| 7 | MT 20 | 60 | 0 | 0 |
| 8 | MT 20 | 60 | 0 | 0 |
| 9 | MT 20 | 60 | 0 | 0 |
| 10 | MT 20 | 120 | 0 | 0 |
| 11 | MT 20 | 120 | 0 | 0 |
| 12 | MT 20 | 120 | 0 | 0 |
| 13 | MT 20 | 120 | 0 | 0 |
| 14 | MT 20 | 120 | 0 | 0 |
| 15 | MT 20 | 120 | 0 | 0 |
| 16 | MT 20 | 120 | 0 | 0 |
| 17 | MT 20 | 120 | 0 | 0 |
| 18 | MT 20 | 120 | 0 | 0 |
| 19 | MT 20 | 120 | 0 | 0 |
| 20 | MT 20 | 120 | 0 | 0 |
| 21 | MT 20 | 120 | 0 | 0 |
| 22 | MT 20 | 120 | 0 | 0 |
| 23 | MT 20 | 120 | 0 | 0 |
| 24 | MT 20 | 120 | 0 | 0 |
| 25 | MT 20 | 120 | 0 | 0 |
| 26 | MT 20 | 120 | 0 | 0 |
| 27 | MT 20 | 120 | 0 | 0 |
| 28 | MT 20 | 120 | 0 | 0 |
| 29 | MT 20 | 120 | 0 | 0 |
| 30 | MT 20 | 120 | 0 | 0 |
| 31 | MT 20 | 120 | 0 | 0 |
| 32 | MT 20 | 120 | 0 | 0 |
| 33 | MT 20 | 120 | 0 | 0 |
| 34 | MT 20 | 120 | 0 | 0 |
|  |  |  |  |  |
| 1 | MS 11 | 120 | 0 | 0 |
| 2 | MS 11 | 120 | 0 | 0 |
| 3 | MS 11 | 120 | 0 | 0 |
| 4 | MS 11 | 120 | 0 | 0 |
| 5 | MS 11 | 120 | 0 | 0 |
| 6 | MS 11 | 120 | 0 | 0 |
| 7 | MS 11 | 120 | 0 | 0 |
| 8 | MS 11 | 120 | 0 | 0 |
| 9 | MS 11 | 120 | 0 | 0 |
| 10 | MS 11 | 120 | 0 | 0 |
| 11 | MS 11 | 120 | 0 | 0 |
| 12 | MS 11 | 120 | 0 | 0 |
| 13 | MS 11 | 120 | 0 | 0 |
| 14 | MS 11 | 120 | 0 | 0 |
| 15 | MS 11 | 120 | 0 | 0 |
| 16 | MS 11 | 120 | 0 | 0 |
| 17 | MS 11 | 120 | 2 | 1 |
| 18 | MS 11 | 120 | 0 | 0 |
| 19 | MS 11 | 120 | 0 | 0 |
| 20 | MS 11 | 120 | 0 | 0 |
| 21 | MS 11 | 120 | 0 | 0 |
| 22 | MS 11 | 120 | 0 | 0 |
| 23 | MS 11 | 120 | 0 | 0 |
| 24 | MS 11 | 120 | 0 | 0 |
| 25 | MS 11 | 120 | 0 | 0 |
| 26 | MS 11 | 120 | 0 | 0 |
| 27 | MS 11 | 120 | 2 | 1 |
| 28 | MS 11 | 120 | 0 | 0 |
| 29 | MS 11 | 120 | 0 | 0 |
| 30 | MS 11 | 120 | 0 | 0 |
| 31 | MS 11 | 120 | 0 | 0 |
| 32 | MS 11 | 120 | 0 | 0 |
| 33 | MS 11 | 120 | 2 | 1 |
| 34 | MS 11 | 120 | 0 | 0 |
| 35 | MS 11 | 120 | 0 | 0 |
| 36 | MS 11 | 120 | 0 | 0 |
| 37 | MS 11 | 120 | 0 | 0 |
| 38 | MS 11 | 120 | 0 | 0 |
| 39 | MS 11 | 120 | 0 | 0 |
| 40 | MS 11 | 120 | 0 | 0 |
| 41 | MS 11 | 120 | 0 | 0 |
| 42 | MS 11 | 120 | 0 | 0 |
| 43 | MS 11 | 120 | 0 | 0 |
| 44 | MS 11 | 120 | 0 | 0 |
| 45 | MS 11 | 120 | 0 | 0 |
| 46 | MS 11 | 120 | 0 | 0 |
| 47 | MS 11 | 120 | 0 | 0 |
| 48 | MS 11 | 120 | 0 | 0 |
| 49 | MS 11 | 120 | 0 | 0 |
| 50 | MS 11 | 120 | 0 | 0 |
| 51 | MS 11 | 120 | 2 | 1 |
|  |  |  |  |  |
| 1 | MS 12 | 60 | 0 | 0 |
| 2 | MS 12 | 120 | 0 | 0 |
| 3 | MS 12 | 120 | 0 | 0 |
| 4 | MS 12 | 120 | 0 | 0 |
| 5 | MS 12 | 120 | 0 | 0 |
| 6 | MS 12 | 120 | 0 | 0 |
| 7 | MS 12 | 120 | 0 | 0 |
| 8 | MS 12 | 120 | 0 | 0 |
| 9 | MS 12 | 120 | 0 | 0 |
| 10 | MS 12 | 120 | 0 | 0 |
| 11 | MS 12 | 120 | 3 | 1 |
| 12 | MS 12 | 120 | 0 | 0 |
| 13 | MS 12 | 120 | 0 | 0 |
| 14 | MS 12 | 120 | 0 | 0 |
| 15 | MS 12 | 120 | 0 | 0 |
| 16 | MS 12 | 120 | 0 | 0 |
| 17 | MS 12 | 120 | 0 | 0 |
| 18 | MS 12 | 120 | 0 | 0 |
| 19 | MS 12 | 120 | 0 | 0 |
| 20 | MS 12 | 120 | 0 | 0 |
| 21 | MS 12 | 120 | 0 | 0 |
|  |  |  |  |  |
| 1 | MS 13 | 120 | 0 | 0 |
| 2 | MS 13 | 120 | 0 | 0 |
| 3 | MS 13 | 120 | 0 | 0 |
| 4 | MS 13 | 120 | 0 | 0 |
| 5 | MS 13 | 120 | 0 | 0 |
| 6 | MS 13 | 120 | 0 | 0 |
| 7 | MS 13 | 120 | 0 | 0 |
| 8 | MS 13 | 120 | 0 | 0 |
| 9 | MS 13 | 120 | 0 | 0 |
| 10 | MS 13 | 120 | 0 | 0 |
| 11 | MS 13 | 120 | 0 | 0 |
| 12 | MS 13 | 120 | 0 | 0 |
| 13 | MS 13 | 120 | 0 | 0 |
| 14 | MS 13 | 120 | 0 | 0 |
| 15 | MS 13 | 120 | 0 | 0 |
| 16 | MS 13 | 120 | 0 | 0 |
| 17 | MS 13 | 120 | 0 | 0 |
| 18 | MS 13 | 120 | 0 | 0 |
| 19 | MS 13 | 120 | 0 | 0 |
| 20 | MS 13 | 120 | 0 | 0 |
| 21 | MS 13 | 120 | 0 | 0 |
| 22 | MS 13 | 120 | 0 | 0 |
| 23 | MS 13 | 120 | 0 | 0 |
| 24 | MS 13 | 120 | 0 | 0 |
| 25 | MS 13 | 120 | 0 | 0 |
| 26 | MS 13 | 120 | 0 | 0 |
| 27 | MS 13 | 120 | 0 | 0 |
|  |  |  |  |  |
| 1 | PR 34 | 120 | 0 | 0 |
| 2 | PR 34 | 120 | 3 | 1 |
| 3 | PR 34 | 120 | 0 | 0 |
| 4 | PR 34 | 120 | 0 | 0 |
| 5 | PR 34 | 40 | 0 | 0 |
| 6 | PR 34 | 120 | 1 | 1 |
| 7 | PR 34 | 120 | 0 | 0 |
| 8 | PR 34 | 120 | 0 | 0 |
| 9 | PR 34 | 120 | 0 | 0 |
| 10 | PR 34 | 120 | 0 | 0 |
| 11 | PR 34 | 120 | 0 | 0 |
| 12 | PR 34 | 120 | 0 | 0 |
| 13 | PR 34 | 120 | 5 | 1 |
| 14 | PR 34 | 120 | 0 | 0 |
| 15 | PR 34 | 120 | 0 | 0 |
| 16 | PR 34 | 120 | 1 | 1 |
| 17 | PR 34 | 120 | 0 | 0 |
| 18 | PR 34 | 120 | 0 | 0 |
| 19 | PR 34 | 120 | 0 | 0 |
| 20 | PR 34 | 120 | 0 | 0 |
| 21 | PR 34 | 120 | 0 | 0 |
| 22 | PR 34 | 120 | 0 | 0 |
| 23 | PR 34 | 120 | 0 | 0 |
| 24 | PR 34 | 120 | 0 | 0 |
| 25 | PR 34 | 120 | 0 | 0 |
| 26 | PR 34 | 120 | 0 | 0 |
| 27 | PR 34 | 120 | 0 | 0 |
| 28 | PR 34 | 120 | 0 | 0 |
| 29 | PR 34 | 120 | 0 | 0 |
| 30 | PR 34 | 120 | 0 | 0 |
| 31 | PR 34 | 120 | 0 | 0 |
| 32 | PR 34 | 60 | 0 | 0 |
| 33 | PR 34 | 120 | 0 | 0 |
| 34 | PR 34 | 120 | 0 | 0 |
| 35 | PR 34 | 40 | 0 | 0 |
| 36 | PR 34 | 120 | 0 | 0 |
| 37 | PR 34 | 120 | 0 | 0 |
| 38 | PR 34 | 120 | 0 | 0 |
| 39 | PR 34 | 60 | 0 | 0 |
| 40 | PR 34 | 120 | 0 | 0 |
| 41 | PR 34 | 80 | 0 | 0 |
| 42 | PR 34 | 120 | 0 | 0 |
| 43 | PR 34 | 120 | 0 | 0 |
| 44 | PR 34 | 80 | 0 | 0 |
| 45 | PR 34 | 120 | 0 | 0 |
| 46 | PR 34 | 120 | 0 | 0 |
| 47 | PR 34 | 120 | 0 | 0 |
| 48 | PR 34 | 120 | 0 | 0 |
| 49 | PR 34 | 120 | 0 | 0 |
| 50 | PR 34 | 120 | 0 | 0 |
| 51 | PR 34 | 120 | 0 | 0 |
| 52 | PR 34 | 120 | 0 | 0 |
| 53 | PR 34 | 120 | 0 | 0 |
| 54 | PR 34 | 120 | 0 | 0 |
| 55 | PR 34 | 120 | 0 | 0 |
| 56 | PR 34 | 100 | 0 | 0 |
| 57 | PR 34 | 40 | 0 | 0 |
| 58 | PR 34 | 120 | 0 | 0 |
| 59 | PR 34 | 120 | 0 | 0 |
| 60 | PR 34 | 120 | 0 | 0 |
| 61 | PR 34 | 120 | 0 | 0 |
| 62 | PR 34 | 120 | 0 | 0 |
| 63 | PR 34 | 120 | 0 | 0 |
| 64 | PR 34 | 120 | 0 | 0 |
| 65 | PR 34 | 120 | 0 | 0 |
| 66 | PR 34 | 120 | 0 | 0 |
| 67 | PR 34 | 120 | 0 | 0 |
| 68 | PR 34 | 120 | 0 | 0 |
| 69 | PR 34 | 120 | 0 | 0 |
|  |  |  |  |  |
| 1 | PR 38 | 120 | 0 | 0 |
| 2 | PR 38 | 120 | 0 | 0 |
| 3 | PR 38 | 120 | 0 | 0 |
| 4 | PR 38 | 120 | 0 | 0 |
| 5 | PR 38 | 120 | 0 | 0 |
| 6 | PR 38 | 120 | 0 | 0 |
| 7 | PR 38 | 120 | 0 | 0 |
| 8 | PR 38 | 120 | 0 | 0 |
| 9 | PR 38 | 120 | 0 | 0 |
| 10 | PR 38 | 120 | 0 | 0 |
| 11 | PR 38 | 120 | 0 | 0 |
| 12 | PR 38 | 120 | 0 | 0 |
| 13 | PR 38 | 120 | 0 | 0 |
| 14 | PR 38 | 120 | 0 | 0 |
| 15 | PR 38 | 120 | 0 | 0 |
| 16 | PR 38 | 120 | 0 | 0 |
| 17 | PR 38 | 120 | 0 | 0 |
| 18 | PR 38 | 120 | 0 | 0 |
| 19 | PR 38 | 120 | 0 | 0 |
| 20 | PR 38 | 120 | 0 | 0 |
| 21 | PR 38 | 120 | 0 | 0 |
| 22 | PR 38 | 120 | 0 | 0 |
| 23 | PR 38 | 120 | 0 | 0 |
| 24 | PR 38 | 120 | 0 | 0 |
| 25 | PR 38 | 120 | 0 | 0 |
| 26 | PR 38 | 80 | 0 | 0 |
| 27 | PR 38 | 120 | 0 | 0 |
| 28 | PR 38 | 120 | 0 | 0 |
| 29 | PR 38 | 120 | 0 | 0 |
| 30 | PR 38 | 120 | 0 | 0 |
| 31 | PR 38 | 120 | 0 | 0 |
| 32 | PR 38 | 120 | 0 | 0 |
| 33 | PR 38 | 120 | 0 | 0 |
| 34 | PR 38 | 120 | 0 | 0 |
| 35 | PR 38 | 120 | 0 | 0 |
| 36 | PR 38 | 80 | 0 | 0 |
| 37 | PR 38 | 120 | 0 | 0 |
| 38 | PR 38 | 120 | 0 | 0 |
| 39 | PR 38 | 120 | 0 | 0 |
