# Supplementary material for: Cross-Resistance between Cry1 Proteins in Fall Armyworm (Spodoptera frugiperda) May Affect the Durability of Current Pyramided Bt Maize Hybrids in Brazil
Source: PLoS One. 2015 Oct 16;10(10):e0140130. doi: 10.1371/journal.pone.0140130 (PMC4608726; doi:10.1371/journal.pone.0140130)
Supplement: S2 Table — (DOCX) [file pone.0140130.s002.docx]

**S2 Table.** Survival of *S. frugiperda* larvae per plant (mean ± SE) on MON 89034 maize and non-Bt near-isoline in greenhouse trials.

| Repetition | RR Strain | |
| --- | --- | --- |
|  | Non-Bt isoline | MON 89034 Isoline |
| 1 | 0 | 1 |
| 2 | 1 | 1 |
| 3 | 1 | 0 |
| 4 | 1 | 1 |
| 5 | 0 | 0 |
| 6 | 1 | 1 |
| 7 | 1 | 0 |
| 8 | 1 | 1 |
| 9 | 1 | 1 |
| 10 | 1 | 1 |
| 11 | 1 | 1 |
| 12 | 1 | 1 |
| 13 | 1 | 0 |
| 14 | 0 | 1 |
| 15 | 0 | 1 |
| 16 | 1 | 1 |
| 17 | 0 | 1 |
| 18 | 1 | 1 |
| 19 | 1 | 0 |
| 20 | 1 | 1 |
| 21 | 0 | 1 |
| 22 | 1 | 1 |
| 23 | 1 | 0 |
| 24 | 1 | 1 |
| 25 | 0 | 0 |
| 26 | 1 | 1 |
| 27 | 1 | 0 |
| 28 | 1 | 1 |
| 29 | 1 | 1 |
| 30 | 1 | 1 |
| 31 | 1 | 1 |
| 32 | 1 | 1 |
| 33 | 1 | 0 |
| 34 | 0 | 1 |
| 35 | 0 | 1 |
| 36 | 1 | 1 |
| 37 | 0 | 1 |
| 38 | 1 | 1 |
| 39 | 1 | 0 |
| 40 | 1 | 1 |
| 41 | 0 | 1 |
| 42 | 1 | 1 |
| 43 | 1 | 0 |
| 44 | 1 | 1 |
| 45 | 0 | 0 |
| 46 | 1 | 1 |
| 47 | 1 | 0 |
| 48 | 1 | 1 |
| 49 | 1 | 1 |
| 50 | 1 | 1 |
| 51 | 1 | 1 |
| 52 | 1 | 1 |
| 53 | 1 | 0 |
| 54 | 1 | 1 |
| 55 | 0 | 1 |
| 56 | 1 | 1 |
| 57 | 0 | 1 |
| 58 | 0 | 1 |
| 59 | 1 | 0 |
| 60 | 1 | 1 |
| 61 | 0 | 1 |
| 62 | 1 | 1 |
| 63 | 1 | 0 |
| 64 | 1 | 1 |
| 65 | 0 | 0 |
| 66 | 1 | 1 |
| 67 | 1 | 0 |
| 68 | 1 | 1 |
| 69 | 1 | 1 |
| 70 | 1 | 1 |
| 71 | 1 | 1 |
| 72 | 1 | 1 |
| 73 | 1 | 1 |
| 74 | 1 | 0 |
| 75 | 0 | 1 |
| 76 | 1 | 0 |
| 77 | 1 | 1 |
| 78 | 1 | 1 |
| 79 | 1 | 1 |
| 80 | 1 | 0 |
| 81 | 1 | 1 |
| 82 | 1 | 1 |
| 83 | 1 | 1 |
| 84 | 1 | 1 |
| 85 | 1 | 1 |
| 86 | 1 | 0 |
| 87 | 1 | 1 |
| 88 | 1 | 1 |
| 89 | 1 | 1 |
| 90 | 1 | 0 |
| 91 | 1 | 1 |
| 92 | 1 | 1 |
| 93 | 1 | 1 |
| 94 | - | - |
| 95 | - | - |
| 96 | - | - |
| 97 | - | - |
| 98 | - | - |
| 99 | - | - |
| 100 | - | - |
| Mean | 0.806451613 | 0.752688172 |
| Standard Deviation | 0.397220356 | 0.433788016 |
| Standard Error | 0.041189832 | 0.044981722 |

| Repetition | SS Strain | |
| --- | --- | --- |
|  | Non-Bt isoline | MON 89034 isoline |
| 1 | 0 | 0 |
| 2 | 0 | 0 |
| 3 | 1 | 0 |
| 4 | 1 | 0 |
| 5 | 1 | 0 |
| 6 | 1 | 0 |
| 7 | 0 | 0 |
| 8 | 1 | 0 |
| 9 | 1 | 0 |
| 10 | 1 | 0 |
| 11 | 1 | 0 |
| 12 | 1 | 0 |
| 13 | 1 | 0 |
| 14 | 1 | 0 |
| 15 | 0 | 0 |
| 16 | 1 | 0 |
| 17 | 0 | 0 |
| 18 | 1 | 0 |
| 19 | 1 | 0 |
| 20 | 1 | 0 |
| 21 | 0 | 0 |
| 22 | 1 | 0 |
| 23 | 1 | 0 |
| 24 | 1 | 0 |
| 25 | 0 | 0 |
| 26 | 1 | 0 |
| 27 | 0 | 0 |
| 28 | 1 | 0 |
| 29 | 1 | 0 |
| 30 | 1 | 0 |
| 31 | 1 | 0 |
| 32 | 1 | 0 |
| 33 | 1 | 0 |
| 34 | 1 | 0 |
| 35 | 0 | 0 |
| 36 | 1 | 0 |
| 37 | 0 | 0 |
| 38 | 1 | 0 |
| 39 | 1 | 0 |
| 40 | 1 | 0 |
| 41 | 0 | 0 |
| 42 | 1 | 0 |
| 43 | 1 | 0 |
| 44 | 1 | 0 |
| 45 | 0 | 0 |
| 46 | 1 | 0 |
| 47 | 0 | 0 |
| 48 | 1 | 0 |
| 49 | 1 | 0 |
| 50 | 1 | 0 |
| 51 | 1 | 0 |
| 52 | 1 | 0 |
| 53 | 1 | 0 |
| 54 | 1 | 0 |
| 55 | 0 | 0 |
| 56 | 1 | 0 |
| 57 | 0 | 0 |
| 58 | 1 | 0 |
| 59 | 1 | 0 |
| 60 | 1 | 0 |
| 61 | 0 | 0 |
| 62 | 1 | 0 |
| 63 | 1 | 0 |
| 64 | 1 | 0 |
| 65 | 0 | 0 |
| 66 | 1 | 0 |
| 67 | 0 | 0 |
| 68 | 1 | 0 |
| 69 | 1 | 0 |
| 70 | 1 | 0 |
| 71 | 1 | 0 |
| 72 | 1 | 0 |
| 73 | 1 | 0 |
| 74 | 1 | 0 |
| 75 | 0 | 0 |
| 76 | 1 | 0 |
| 77 | 0 | 0 |
| 78 | 1 | 0 |
| 79 | 1 | 0 |
| 80 | 1 | 0 |
| 81 | 0 | 0 |
| 82 | 1 | 0 |
| 83 | 1 | 0 |
| 84 | 1 | 0 |
| 85 | 0 | 0 |
| 86 | 1 | 0 |
| 87 | 0 | 0 |
| 88 | 1 | 0 |
| 89 | 1 | 0 |
| 90 | 1 | 0 |
| 91 | 1 | 0 |
| 92 | 1 | 0 |
| 93 | 1 | 0 |
| 94 | 1 | 0 |
| 95 | 0 | 0 |
| 96 | 1 | 0 |
| 97 | 1 | 0 |
| 98 | 1 | 0 |
| 99 | 1 | 0 |
| 100 | 0 | 0 |
| Mean | 0.75 | 0 |
| Standard Deviation | 0.43519414 | 0 |
| Standard Error | 0.043519414 | 0 |

| Repetition | | S♂R♀ Strain | | | |
| --- | --- | --- | --- | --- | --- |
|  |  | Non-Bt isoline | | MON 89034 isoline | |
| 1 | | 1 | | 0 | |
| 2 | | 1 | | 0 | |
| 3 | | 1 | | 0 | |
| 4 | | 1 | | 0 | |
| 5 | | 0 | | 0 | |
| 6 | | 1 | | 0 | |
| 7 | | 1 | | 0 | |
| 8 | | 1 | | 0 | |
| 9 | | 1 | | 0 | |
| 10 | | 1 | | 0 | |
| 11 | | 1 | | 0 | |
| 12 | | 1 | | 0 | |
| 13 | | 0 | | 0 | |
| 14 | | 0 | | 0 | |
| 15 | | 0 | | 0 | |
| 16 | | 1 | | 0 | |
| 17 | | 0 | | 0 | |
| 18 | | 1 | | 0 | |
| 19 | | 1 | | 0 | |
| 20 | | 1 | | 0 | |
| 21 | | 1 | | 0 | |
| 22 | | 1 | | 0 | |
| 23 | | 1 | | 0 | |
| 24 | | 1 | | 0 | |
| 25 | | 0 | | 0 | |
| 26 | | 1 | | 0 | |
| 27 | | 1 | | 0 | |
| 28 | | 1 | | 0 | |
| 29 | | 1 | | 0 | |
| 30 | | 1 | | 0 | |
| 31 | | 1 | | 0 | |
| 32 | | 1 | | 0 | |
| 33 | | 0 | | 0 | |
| 34 | | 0 | | 0 | |
| 35 | | 0 | | 0 | |
| 36 | | 1 | | 0 | |
| 37 | | 0 | | 0 | |
| 38 | | 1 | | 0 | |
| 39 | | 1 | | 0 | |
| 40 | | 1 | | 0 | |
| 41 | | 1 | | 0 | |
| 42 | | 1 | | 0 | |
| 43 | | 1 | | 0 | |
| 44 | | 1 | | 0 | |
| 45 | | 0 | | 0 | |
| 46 | | 1 | | 0 | |
| 47 | | 1 | | 0 | |
| 48 | | 1 | | 0 | |
| 49 | | 1 | | 0 | |
| 50 | | 1 | | 0 | |
| 51 | | 1 | | 0 | |
| 52 | | 1 | | 0 | |
| 53 | | 0 | | 0 | |
| 54 | | 0 | | 0 | |
| 55 | | 0 | | 0 | |
| 56 | | 1 | | 0 | |
| 57 | | 0 | | 0 | |
| 58 | | 1 | | 0 | |
| 59 | | 1 | | 0 | |
| 60 | | 1 | | 0 | |
| 61 | | 1 | | 0 | |
| 62 | | 1 | | 0 | |
| 63 | | 1 | | 0 | |
| 64 | | 1 | | 0 | |
| 65 | | 0 | | 0 | |
| 66 | | 1 | | 0 | |
| 67 | | 1 | | 0 | |
| 68 | | 1 | | 0 | |
| 69 | | 1 | | 0 | |
| 70 | | 1 | | 0 | |
| 71 | | 1 | | 0 | |
| 72 | | 1 | | 0 | |
| 73 | | 0 | | 0 | |
| 74 | | 0 | | 0 | |
| 75 | | 0 | | 0 | |
| 76 | | 1 | | 0 | |
| 77 | | 0 | | 0 | |
| 78 | | 1 | | 0 | |
| 79 | | 1 | | 0 | |
| 80 | | 1 | | 0 | |
| 81 | | 1 | | 0 | |
| 82 | | 1 | | 0 | |
| 83 | | 1 | | 0 | |
| 84 | | 1 | | 0 | |
| 85 | | 1 | | 0 | |
| 86 | | 1 | | 0 | |
| 87 | | 1 | | 0 | |
| 88 | | 1 | | 0 | |
| 89 | | 1 | | 0 | |
| 90 | | 1 | | 0 | |
| 91 | | 1 | | 0 | |
| 92 | | 1 | | 0 | |
| 93 | | 1 | | 0 | |
| 94 | | 1 | | 0 | |
| 95 | | 1 | | 0 | |
| 96 | | 1 | | 0 | |
| 97 | | 1 | | 0 | |
| 98 | | 1 | | 0 | |
| 99 | | 1 | | 0 | |
| 100 | | 1 | | 0 | |
| Mean | | 0.8 | | 0 | |
| Standard Deviation | | 0.402015126 | | 0 | |
| Standard Error | | 0.040201513 | | 0 | |
|  | |  | |  | |
| Repetition | S♀R♂ Strain | | | |  |
|  | Non-Bt isoline | | MON 89034 isoline | |  |
| 1 | 0 | | 0 | |  |
| 2 | 1 | | 0 | |  |
| 3 | 1 | | 0 | |  |
| 4 | 1 | | 0 | |  |
| 5 | 0 | | 0 | |  |
| 6 | 1 | | 0 | |  |
| 7 | 1 | | 0 | |  |
| 8 | 0 | | 0 | |  |
| 9 | 0 | | 0 | |  |
| 10 | 1 | | 0 | |  |
| 11 | 1 | | 0 | |  |
| 12 | 1 | | 0 | |  |
| 13 | 1 | | 0 | |  |
| 14 | 1 | | 0 | |  |
| 15 | 1 | | 0 | |  |
| 16 | 1 | | 0 | |  |
| 17 | 1 | | 0 | |  |
| 18 | 1 | | 0 | |  |
| 19 | 1 | | 0 | |  |
| 20 | 1 | | 0 | |  |
| 21 | 0 | | 0 | |  |
| 22 | 1 | | 0 | |  |
| 23 | 1 | | 0 | |  |
| 24 | 1 | | 0 | |  |
| 25 | 0 | | 0 | |  |
| 26 | 1 | | 0 | |  |
| 27 | 1 | | 0 | |  |
| 28 | 1 | | 0 | |  |
| 29 | 1 | | 0 | |  |
| 30 | 1 | | 0 | |  |
| 31 | 1 | | 0 | |  |
| 32 | 1 | | 0 | |  |
| 33 | 1 | | 0 | |  |
| 34 | 1 | | 0 | |  |
| 35 | 0 | | 0 | |  |
| 36 | 1 | | 0 | |  |
| 37 | 0 | | 0 | |  |
| 38 | 1 | | 0 | |  |
| 39 | 1 | | 0 | |  |
| 40 | 1 | | 0 | |  |
| 41 | 0 | | 0 | |  |
| 42 | 1 | | 0 | |  |
| 43 | 1 | | 0 | |  |
| 44 | 1 | | 0 | |  |
| 45 | 0 | | 0 | |  |
| 46 | 1 | | 0 | |  |
| 47 | 1 | | 0 | |  |
| 48 | 1 | | 0 | |  |
| 49 | 1 | | 0 | |  |
| 50 | 1 | | 0 | |  |
| 51 | 1 | | 0 | |  |
| 52 | 1 | | 0 | |  |
| 53 | 1 | | 0 | |  |
| 54 | 1 | | 0 | |  |
| 55 | 0 | | 0 | |  |
| 56 | 1 | | 0 | |  |
| 57 | 0 | | 0 | |  |
| 58 | 1 | | 0 | |  |
| 59 | 1 | | 0 | |  |
| 60 | 1 | | 0 | |  |
| 61 | 0 | | 0 | |  |
| 62 | 1 | | 0 | |  |
| 63 | 1 | | 0 | |  |
| 64 | 1 | | 0 | |  |
| 65 | 0 | | 0 | |  |
| 66 | 1 | | 0 | |  |
| 67 | 1 | | 0 | |  |
| 68 | 1 | | 0 | |  |
| 69 | 1 | | 0 | |  |
| 70 | 1 | | 0 | |  |
| 71 | 1 | | 0 | |  |
| 72 | 1 | | 0 | |  |
| 73 | 1 | | 0 | |  |
| 74 | 1 | | 0 | |  |
| 75 | 1 | | 0 | |  |
| 76 | 1 | | 0 | |  |
| 77 | 1 | | 0 | |  |
| 78 | 1 | | 0 | |  |
| 79 | 1 | | 0 | |  |
| 80 | 1 | | 0 | |  |
| 81 | 1 | | 0 | |  |
| 82 | 1 | | 0 | |  |
| 83 | 1 | | 0 | |  |
| 84 | 1 | | 0 | |  |
| 85 | 1 | | 0 | |  |
| 86 | 1 | | 0 | |  |
| 87 | 1 | | 0 | |  |
| 88 | 1 | | 0 | |  |
| 89 | 1 | | 0 | |  |
| 90 | 1 | | 0 | |  |
| 91 | 1 | | 0 | |  |
| 92 | 1 | | 0 | |  |
| 93 | 1 | | 0 | |  |
| 94 | 1 | | 0 | |  |
| 95 | 1 | | 0 | |  |
| 96 | 1 | | 0 | |  |
| 97 | 1 | | 0 | |  |
| 98 | - | | - | |  |
| 99 | - | | - | |  |
| 100 | - | | - | |  |
| Mean | 0.855670103 | | 0 | |  |
| Standard Deviation | 0.35324952 | | 0 | |  |
| Standard Error | 0.035867055 | | 0 | |  |
