# Supplementary material for: Cross-Resistance between Cry1 Proteins in Fall Armyworm (Spodoptera frugiperda) May Affect the Durability of Current Pyramided Bt Maize Hybrids in Brazil
Source: PLoS One. 2015 Oct 16;10(10):e0140130. doi: 10.1371/journal.pone.0140130 (PMC4608726; doi:10.1371/journal.pone.0140130)
Supplement: S3 Table — (DOCX) [file pone.0140130.s003.docx]

**S3 Table.** Concentration-response and growth inhibition response (MIC_50_ and EC_50_; ng/cm^2^) of *S. frugiperda* in diet-overlay bioassays with purified Cry1A.105 and Cry2Ab2.

| **Cry1A.105_protein_RR Strain** | | | | | | | | | | | | | | |
| --- | --- | --- | --- | --- | --- | --- | --- | --- | --- | --- | --- | --- | --- | --- |
| Number of tested larvae (T) and mortality (M) | | | | | | | | | | | | | | |
| Concentration  (ng/cm2) | Mortality | | | | | | | | | | | | TOTAL | |
|  | Repetition 1 | | Repetition 2 | | Repetition 3 | | Repetition 4 | | Repetition 5 | | Repetition 6 | |  |  |
|  | T | M | T | M | T | M | T | M | T | M | T | M | T | M |
| 0 | 16 | 0 | 13 | 0 | 16 | 0 | 16 | 0 | - | - | - | - | 61 | 0 |
| 508.736 | 15 | 0 | 15 | 2 | 16 | 3 | 16 | 3 | - | - | - | - | 62 | 8 |
| 890.288 | 16 | 0 | 16 | 2 | 16 | 2 | 16 | 2 | 16 | 0 | - | - | 80 | 6 |
| 1589.8 | 16 | 1 | 16 | 1 | 15 | 1 | 16 | 1 | 16 | 1 | - | - | 79 | 5 |
| 5087.36 | 15 | 3 | 15 | 3 | 16 | 3 | 16 | 2 | 16 | 3 | - | - | 78 | 14 |
| 8902.88 | 15 | 7 | 16 | 7 | 16 | 7 | 13 | 6 | 15 | 7 | - | - | 75 | 34 |
| 15898 | 16 | 8 | 16 | 6 | 16 | 9 | 16 | 8 | 16 | 9 | 16 | 6 | 80 | 46 |
|  |  | | | | | | | | | | | | | |
| Concentration  (ng/cm2) | Inhibition response (mg) | | | | | | | | | | | | TOTAL | |
|  | Repetition 1 | | Repetition 2 | | Repetition 3 | | Repetition 4 | | Repetition 5 | | Repetition 6 | |  |  |
|  | NWL | P | NWL | P | NWL | P | NWL | P | NWL | P | NWL | P | NWL | P |
| 0 | 16 | 299.3 | 13 | 258.3 | 16 | 256.3 | 16 | 328.3 | - | - | - | - | 61 | 1142.2 |
| 508.736 | 15 | 251.2 | 13 | 209.3 | 13 | 210.1 | 13 | 235.5 | - | - | - | - | 54 | 906.1 |
| 890.288 | 16 | 203.4 | 14 | 207.7 | 14 | 172.0 | 16 | 171.1 | - | - | - | - | 60 | 754.2 |
| 1589.8 | 15 | 199.4 | 15 | 174.4 | 15 | 152.3 | 15 | 180.3 | 15 | 186.3 | - | - | 75 | 892.7 |
| 5087.36 | 12 | 94.5 | 12 | 99.0 | 13 | 93.0 | 12 | 80.4 | 13 | 95.2 | - | - | 62 | 462.1 |
| 8902.88 | 8 | 72.0 | 9 | 73.7 | 9 | 78.0 | 7 | 75.8 | 8 | 71.5 | - | - | 41 | 371.0 |
| 15898 | 8 | 71.5 | 10 | 72.5 | 7 | 72.1 | 10 | 39.1 | 7 | 70.5 | 10 | 70.2 | 52 | 395.9 |
| NWL: Number of weighed larvae | | | | | |  |  |  |  |  |  |  |  |  |
| P: Total weight (mg) | | | | | |  |  |  |  |  |  |  |  |  |

| **Cry1A.105_protein_S♀r♂ Strain** | | | | | | | | | | |
| --- | --- | --- | --- | --- | --- | --- | --- | --- | --- | --- |
| Number of tested larvae (T) and mortality (M) | | | | | | | | | | |
| Concentration  (ng/cm2) | Mortality | | | | | | | | TOTAL | |
|  | Repetition 1 | | Repetition 2 | | Repetition 3 | | Repetition 4 | |  |  |
|  | T | M | T | M | T | M | T | M | T | M |
| 0 | 16 | 0 | 16 | 0 | 16 | 0 | 16 | 0 | 64 | 0 |
| 1.5898 | 16 | 2 | 16 | 1 | 16 | 1 | 16 | 2 | 64 | 6 |
| 5.08736 | 16 | 5 | 16 | 5 | 16 | 5 | 16 | 5 | 64 | 20 |
| 15.898 | 16 | 7 | 16 | 7 | 16 | 6 | 16 | 7 | 64 | 27 |
| 50.8736 | 16 | 9 | 16 | 10 | 16 | 10 | 16 | 9 | 64 | 38 |
| 158.98 | 16 | 11 | 16 | 12 | 16 | 12 | 16 | 11 | 64 | 46 |
| 508.736 | 16 | 12 | 16 | 13 | 16 | 13 | 16 | 13 | 64 | 51 |
| 1589.8 | 16 | 14 | 16 | 13 | 16 | 14 | 16 | 15 | 64 | 56 |
| 5087.36 | 16 | 15 | 16 | 16 | 16 | 15 | 16 | 16 | 64 | 62 |
|  |  | | | | | | | | | |
| Concentration  (ng/cm2) | Inhibition response (mg) | | | | | | | | TOTAL | |
|  | Repetition 1 | | Repetition 2 | | Repetition 3 | | Repetition 4 | |  |  |
|  | NWL | P | NWL | P | NWL | P | NWL | P | NWL | P |
| 0 | 16 | 354.1 | 16 | 331.9 | 16 | 350.1 | 16 | 369.2 | 64 | 1405.3 |
| 1.5898 | 14 | 255.6 | 15 | 257.5 | 15 | 273.1 | 14 | 286.1 | 58 | 1072.3 |
| 5.08736 | 11 | 163.5 | 11 | 197.5 | 11 | 185.1 | 10 | 196.4 | 43 | 742.5 |
| 15.898 | 9 | 101.3 | 9 | 102.9 | 10 | 104.6 | 9 | 105.6 | 37 | 414.4 |
| 50.8736 | 7 | 46.2 | 6 | 48.2 | 6 | 47.4 | 9 | 46.1 | 28 | 187.9 |
| 158.98 | 5 | 24.1 | 4 | 23.1 | 4 | 22.8 | 5 | 27.3 | 18 | 97.3 |
| 508.736 | 4 | 11.0 | 3 | 15.2 | 3 | 12.4 | 4 | 11.9 | 14 | 50.5 |
| 1589.8 | 2 | 4.1 | 3 | 4.8 | 2 | 4.9 | 1 | 4.7 | 8 | 18.5 |
| 5087.36 | 1 | 1 | 0 | 0 | 1 | 0.75 | 0 | 0 | 2 | 1.75 |
| NWL: Number of weighed larvae | | | | | |  |  |  |  |  |
| P: Total weight (mg) | | | | | |  |  |  |  |  |

| **Cry1A.105_protein_S♂R♀ Strain** | | | | | | | | | | |
| --- | --- | --- | --- | --- | --- | --- | --- | --- | --- | --- |
| Number of tested larvae (T) and mortality (M) | | | | | | | | | | |
| Concentration  (ng/cm2) | Mortality | | | | | | | | TOTAL | |
|  | Repetition 1 | | Repetition 2 | | Repetition 3 | | Repetition 3 | |  |  |
|  | T | M | T | M | T | M | T | M | T | M |
| 0 | 16 | 0 | 16 | 0 | 16 | 0 | 16 | 0 | 64 | 0 |
| 1.5898 | 16 | 2 | 16 | 2 | 16 | 2 | 16 | 2 | 64 | 8 |
| 5.08736 | 16 | 5 | 16 | 5 | 16 | 5 | 16 | 6 | 64 | 21 |
| 15.898 | 16 | 7 | 16 | 7 | 16 | 8 | 16 | 8 | 64 | 30 |
| 50.8736 | 16 | 11 | 16 | 10 | 16 | 10 | 16 | 11 | 64 | 42 |
| 158.98 | 16 | 11 | 16 | 12 | 16 | 12 | 16 | 11 | 64 | 46 |
| 508.736 | 16 | 12 | 16 | 13 | 16 | 13 | 16 | 12 | 64 | 50 |
| 1589.8 | 16 | 14 | 16 | 14 | 16 | 14 | 16 | 15 | 64 | 57 |
| 5087.36 | 16 | 15 | 16 | 16 | 16 | 15 | 16 | 16 | 64 | 62 |
|  |  | | | | | | | | | |
| Concentration  (ng/cm2) | Inhibition response (mg) | | | | | | | | TOTAL | |
|  | Repetition 1 | | Repetition 2 | | Repetition 3 | | Repetition 4 | |  |  |
|  | NWL | P | NWL | P | NWL | P | NWL | P | NWL | P |
| 0 | 16 | 334.1 | 16 | 351.7 | 16 | 347.1 | 16 | 349.2 | 64 | 1382.1 |
| 1.5898 | 14 | 170.6 | 14 | 196.5 | 14 | 193.1 | 14 | 196.9 | 56 | 757.1 |
| 5.08736 | 11 | 114.5 | 11 | 115.0 | 11 | 117.1 | 10 | 118.4 | 43 | 465.0 |
| 15.898 | 9 | 89.3 | 9 | 82.9 | 8 | 81.6 | 8 | 85.0 | 34 | 338.8 |
| 50.8736 | 5 | 56.2 | 6 | 58.2 | 6 | 57.4 | 5 | 56.1 | 22 | 227.9 |
| 158.98 | 5 | 22.1 | 4 | 24.1 | 4 | 23.9 | 5 | 20.3 | 18 | 90.4 |
| 508.736 | 4 | 13.0 | 3 | 13.2 | 3 | 11.6 | 4 | 11.9 | 14 | 49.7 |
| 1589.8 | 2 | 4.1 | 2 | 4.8 | 2 | 4.9 | 1 | 4.7 | 7 | 18.5 |
| 5087.36 | 1 | 0.8 | 0 | 0 | 1 | 0.6 | 0 | 0 | 2 | 1.4 |
| NWL: Number of weighed larvae | | | | | |  |  |  |  |  |
| P: Total weight (mg) | | | | | |  |  |  |  |  |

| **Cry2Ab2_protein_RR Strain** | | | | | | | | | | |
| --- | --- | --- | --- | --- | --- | --- | --- | --- | --- | --- |
| Number of tested larvae (T) and mortality (M) | | | | | | | | | | |
| Concentration  (ng/cm2) | Mortality | | | | | | | | TOTAL | |
|  | Repetition 1 | | Repetition 2 | | Repetition 3 | | Repetition 4 | |  |  |
|  | T | M | T | M | T | M | T | M | T | M |
| 0 | 16 | 0 | 15 | 0 | 16 | 0 | 16 | 0 | 63 | 0 |
| 5.08736 | 16 | 2 | 16 | 2 | 16 | 2 | 16 | 1 | 64 | 7 |
| 15.898 | 16 | 4 | 16 | 4 | 16 | 4 | 16 | 3 | 64 | 15 |
| 50.8736 | 16 | 7 | 16 | 7 | 16 | 7 | 16 | 6 | 64 | 27 |
| 158.98 | 16 | 9 | 16 | 9 | 16 | 9 | 15 | 9 | 63 | 36 |
| 508.736 | 16 | 10 | 16 | 10 | 16 | 10 | 16 | 11 | 64 | 41 |
| 1589.8 | 14 | 12 | 16 | 12 | 16 | 12 | 16 | 12 | 62 | 48 |
| 4976.074 | 16 | 14 | 16 | 14 | 16 | 12 | 16 | 12 | 64 | 52 |
|  |  | | | | | | | | | |
| Concentration  (ng/cm2) | Inhibition response (mg) | | | | | | | | TOTAL | |
|  | Repetition 1 | | Repetition 2 | | Repetition 3 | | Repetition 4 | |  |  |
|  | NWL | P | NWL | P | NWL | P | NWL | P | NWL | P |
| 0 | 16 | 285.1 | 15 | 320.8 | 16 | 383.8 | 16 | 261.5 | 63 | 1251.2 |
| 5.08736 | 14 | 175.9 | 14 | 174.1 | 14 | 171.9 | 15 | 160.6 | 57 | 682.5 |
| 15.898 | 12 | 121.3 | 12 | 125.5 | 12 | 128.9 | 13 | 128.0 | 49 | 503.7 |
| 50.8736 | 9 | 69.9 | 9 | 64.9 | 9 | 67.9 | 10 | 62.2 | 37 | 264.9 |
| 158.98 | 7 | 42.8 | 7 | 45.1 | 7 | 47.1 | 7 | 48.4 | 28 | 183.4 |
| 508.736 | 6 | 31.1 | 6 | 38.2 | 6 | 35.1 | 5 | 34.0 | 23 | 138.4 |
| 1589.8 | 4 | 20.5 | 4 | 21.9 | 4 | 21.3 | 4 | 21.1 | 16 | 84.8 |
| 4976.074 | 2 | 8.0 | 2 | 6.1 | 4 | 11.8 | 4 | 11.1 | 12 | 37.0 |
| NWL: Number of weighed larvae | | | | | |  |  |  |  |  |
| P: Total weight (mg) | | | | | |  |  |  |  |  |

| **Cry2Ab2_protein_SS Strain** | | | | | | | | | | |
| --- | --- | --- | --- | --- | --- | --- | --- | --- | --- | --- |
| Number of tested larvae (T) and mortality (M) | | | | | | | | | | |
| Concentration  (ng/cm2) | Mortality | | | | | | | | TOTAL | |
|  | Repetition 1 | | Repetition 2 | | Repetition 3 | | Repetition 4 | |  |  |
|  | T | M | T | M | T | M | T | M | T | M |
| 0 | 16 | 0 | 15 | 0 | 16 | 0 | 16 | 0 | 63 | 0 |
| 0.508736 | 16 | 1 | 16 | 2 | 16 | 1 | 16 | 1 | 64 | 5 |
| 1.5898 | 16 | 4 | 16 | 3 | 16 | 3 | 16 | 3 | 64 | 13 |
| 5.08736 | 16 | 6 | 16 | 7 | 16 | 6 | 16 | 6 | 64 | 25 |
| 15.898 | 16 | 10 | 16 | 10 | 15 | 9 | 16 | 9 | 63 | 38 |
| 50.8736 | 16 | 11 | 16 | 11 | 16 | 11 | 16 | 11 | 64 | 44 |
| 158.98 | 16 | 12 | 16 | 12 | 16 | 12 | 16 | 12 | 64 | 48 |
| 508.736 | 16 | 13 | 16 | 14 | 16 | 14 | 16 | 14 | 64 | 55 |
| 1589.8 | 16 | 15 | 16 | 15 | 16 | 15 | 15 | 15 | 63 | 60 |
| 2861.64 | 16 | 16 | 16 | 16 | 16 | 16 | 15 | 15 | 63 | 63 |
|  |  |  |  |  |  |  |  |  |  |  |
| Concentration  (ng/cm2) | Inhibition response (mg) | | | | | | | | TOTAL | |
|  | Repetition 1 | | Repetition 2 | | Repetition 3 | | Repetition 4 | |  |  |
|  | NWL | P | NWL | P | NWL | P | NWL | P | NWL | P |
| 0 | 16 | 700.1 | 15 | 661.1 | 16 | 663.3 | 16 | 671.3 | 63 | 2695.8 |
| 0.508736 | 15 | 440.1 | 14 | 461.1 | 15 | 463.3 | 15 | 471.3 | 59 | 1835.8 |
| 1.5898 | 12 | 299.2 | 13 | 297.2 | 13 | 298.3 | 13 | 290.5 | 51 | 1185.2 |
| 5.08736 | 10 | 179.4 | 9 | 178.5 | 10 | 175.1 | 10 | 173.3 | 39 | 706.3 |
| 15.898 | 6 | 100.9 | 6 | 105.9 | 7 | 108.6 | 7 | 108.6 | 26 | 424.0 |
| 50.8736 | 5 | 65.8 | 5 | 67.6 | 5 | 60.6 | 5 | 69.1 | 20 | 263.1 |
| 158.98 | 4 | 42.0 | 4 | 46.8 | 4 | 40.1 | 4 | 40.1 | 16 | 169.0 |
| 508.736 | 3 | 16.1 | 2 | 13.5 | 2 | 11.6 | 2 | 13.8 | 9 | 55.0 |
| 1589.8 | 1 | 2.7 | 1 | 3.0 | 1 | 3.1 | 0 | 0.0 | 3 | 8.8 |
| 2861.64 | 0 | 0.0 | 0 | 0.0 | 0 | 0.0 | 0 | 0.0 | 0 | 0.0 |
| NWL: Number of weighed larvae | | | | | |  |  |  |  |  |
| P: Total weight (mg) | | | | | |  |  |  |  |  |

| **Cry2Ab2_protein_S♀R♂ Strain** | | | | | | | | | | |
| --- | --- | --- | --- | --- | --- | --- | --- | --- | --- | --- |
| Number of tested larvae (T) and mortality (M) | | | | | | | | | | |
| Concentration  (ng/cm2) | Mortality | | | | | | | | TOTAL | |
|  | Repetition 1 | | Repetition 2 | | Repetition 3 | | Repetition 4 | |  |  |
|  | T | M | T | M | T | M | T | M | T | M |
| 0 | 16 | 0 | 16 | 0 | 16 | 0 | 16 | 0 | 64 | 0 |
| 0.508736 | 16 | 0 | 16 | 0 | 16 | 1 | 16 | 1 | 64 | 2 |
| 1.5898 | 16 | 2 | 16 | 1 | 16 | 2 | 16 | 2 | 64 | 7 |
| 5.08736 | 16 | 3 | 16 | 3 | 16 | 2 | 16 | 3 | 64 | 11 |
| 15.898 | 16 | 5 | 16 | 4 | 16 | 6 | 16 | 5 | 64 | 20 |
| 50.8736 | 16 | 10 | 16 | 11 | 16 | 11 | 16 | 10 | 64 | 42 |
| 158.98 | 16 | 11 | 16 | 11 | 16 | 11 | 16 | 11 | 64 | 44 |
| 508.736 | 16 | 12 | 16 | 11 | 16 | 12 | 16 | 11 | 64 | 46 |
| 1589.8 | 16 | 14 | 16 | 14 | 16 | 14 | 16 | 15 | 64 | 57 |
| 2861.64 | 16 | 16 | 16 | 16 | 16 | 16 | 16 | 16 | 64 | 64 |
|  |  |  |  |  |  |  |  |  |  |  |
| Concentration  (ng/cm2) | Inhibition response (mg) | | | | | | | | TOTAL | |
|  | Repetition 1 | | Repetition 2 | | Repetition 3 | | Repetition 14 | |  |  |
|  | NWL | P | NWL | P | NWL | P | NWL | P | NWL | P |
| 0 | 16 | 210.7 | 16 | 211.9 | 16 | 200.4 | 16 | 235.1 | 64 | 858.1 |
| 0.508736 | 16 | 137.4 | 15 | 161.5 | 14 | 154.8 | 14 | 145.6 | 59 | 599.3 |
| 1.5898 | 14 | 121.8 | 15 | 117.5 | 14 | 119.4 | 14 | 111.7 | 57 | 470.4 |
| 5.08736 | 13 | 92.5 | 13 | 99.3 | 14 | 100.4 | 13 | 98.5 | 53 | 390.7 |
| 15.898 | 11 | 61.2 | 12 | 65.1 | 10 | 60.1 | 11 | 62.5 | 44 | 248.9 |
| 50.8736 | 6 | 28.7 | 5 | 28.8 | 5 | 24.9 | 6 | 25.6 | 22 | 108.0 |
| 158.98 | 5 | 13.1 | 5 | 11.3 | 5 | 10.0 | 5 | 10.1 | 20 | 44.5 |
| 508.736 | 4 | 4.8 | 5 | 4.3 | 4 | 4.1 | 5 | 4.5 | 18 | 17.7 |
| 1589.8 | 2 | 1.3 | 2 | 1.0 | 2 | 1.1 | 1 | 1.9 | 7 | 5.3 |
| 2861.64 | 0 | 0.0 | 0 | 0.0 | 0 | 0.0 | 0 | 0.0 | 0 | 0.0 |
| NWL: Number of weighed larvae | | | | | |  |  |  |  |  |
| P: Total weight (mg) | | | | | |  |  |  |  |  |

| **Cry2Ab2_protein_S♂R♀ Strain** | | | | | | | | | | |
| --- | --- | --- | --- | --- | --- | --- | --- | --- | --- | --- |
| Number of tested larvae (T) and mortality (M) | | | | | | | | | | |
| Concentration  (ng/cm2) | Mortality | | | | | | | | TOTAL | |
|  | Repetition 1 | | Repetition 2 | | Repetition 3 | | Repetition 4 | |  |  |
|  | T | M | T | M | T | M | T | M | T | M |
| 0 | 16 | 0 | 16 | 0 | 16 | 0 | 16 | 0 | 64 | 0 |
| 0.508736 | 16 | 0 | 16 | 0 | 16 | 1 | 16 | 1 | 64 | 2 |
| 1.5898 | 16 | 2 | 16 | 3 | 16 | 3 | 16 | 2 | 64 | 10 |
| 5.08736 | 16 | 3 | 16 | 3 | 16 | 2 | 16 | 1 | 64 | 9 |
| 15.898 | 16 | 5 | 16 | 5 | 16 | 6 | 16 | 5 | 64 | 21 |
| 50.8736 | 16 | 10 | 16 | 10 | 16 | 10 | 16 | 10 | 64 | 40 |
| 158.98 | 16 | 11 | 16 | 11 | 16 | 11 | 16 | 11 | 64 | 44 |
| 508.736 | 16 | 12 | 16 | 11 | 16 | 11 | 16 | 11 | 64 | 45 |
| 1589.8 | 16 | 14 | 16 | 14 | 16 | 14 | 16 | 13 | 64 | 55 |
| 2861.64 | 16 | 16 | 16 | 16 | 16 | 16 | 16 | 15 | 64 | 63 |
|  |  |  |  |  |  |  |  |  |  |  |
| Concentration  (ng/cm2) | Inhibition response (mg) | | | | | | | | TOTAL | |
|  | Repetition 1 | | Repetition 2 | | Repetition 3 | | Repetition 4 | |  |  |
|  | NWL | P | NWL | P | NWL | P | NWL | P | NWL | P |
| 0 | 16 | 210.7 | 16 | 211.9 | 16 | 200.4 | 16 | 235.1 | 64 | 858.1 |
| 0.508736 | 16 | 137.4 | 15 | 161.5 | 15 | 154.8 | 15 | 145.6 | 61 | 599.3 |
| 1.5898 | 14 | 121.8 | 13 | 117.5 | 13 | 119.4 | 14 | 111.7 | 54 | 470.4 |
| 5.08736 | 13 | 92.5 | 13 | 99.3 | 14 | 100.4 | 15 | 98.5 | 55 | 390.7 |
| 15.898 | 11 | 71.2 | 11 | 75.1 | 10 | 70.1 | 11 | 72.5 | 43 | 288.9 |
| 50.8736 | 6 | 25.7 | 6 | 30.8 | 6 | 31.9 | 6 | 29.6 | 24 | 118.0 |
| 158.98 | 5 | 13.1 | 5 | 11.3 | 5 | 10.0 | 5 | 10.1 | 20 | 44.5 |
| 508.736 | 4 | 5.8 | 5 | 6.3 | 5 | 7.1 | 5 | 5.5 | 19 | 24.7 |
| 1589.8 | 2 | 2.3 | 2 | 2.0 | 2 | 2.1 | 3 | 1.9 | 9 | 8.3 |
| 2861.64 | 0 | 0.0 | 0 | 0.0 | 0 | 0.0 | 1 | 0.6 | 1 | 0.6 |
| NWL: Number of weighed larvae | | | | | |  |  |  |  |  |
| P: Total weight (mg) | | | | | |  |  |  |  |  |
