# Supplementary material for: Cross-Resistance between Cry1 Proteins in Fall Armyworm (Spodoptera frugiperda) May Affect the Durability of Current Pyramided Bt Maize Hybrids in Brazil
Source: PLoS One. 2015 Oct 16;10(10):e0140130. doi: 10.1371/journal.pone.0140130 (PMC4608726; doi:10.1371/journal.pone.0140130)
Supplement: S4 Table — (DOCX) [file pone.0140130.s004.docx]

**S4 Table.** Concentration-response of *S. frugiperda* in diet-overlay bioassays with purified Cry1A.105 and Cry2Ab2 proteins.

| Cry1A.105 protein_ SS Strain | | | | |
| --- | --- | --- | --- | --- |
| Concentration (ng/cm^2^) | Corrected mortality (%) | SE | % Growth | SE |
| 0.0 | 0.0 | 0.0 | 0.0 | 4.9 |
| 0.2 | 7.8 | 2.6 | 56.9 | 0.9 |
| 0.5 | 20.3 | 3.6 | 63.2 | 1.3 |
| 1.6 | 33.9 | 2.1 | 60.2 | 2.1 |
| 5.1 | 37.5 | 3.2 | 81.7 | 0.2 |
| 15.9 | 65.6 | 3.8 | 77.2 | 0.6 |
| 50.9 | 93.1 | 2.9 | 79.3 | 1.2 |
| 159.0 | 93.8 | 2.6 | 80.8 | 4.8 |
| 508.7 | 96.9 | 1.8 | 94.1 | 0.7 |
|  |  |  |  |  |
| Cry1A.105 protein_RR Strain | | | | |
| Concentration (ng/cm^2^) | Corrected mortality (%) | SE | % Growth | SE |
| 0.0 | 0.0 | 0.0 | 0.0 | 0.0 |
| 508.7 | 12.9 | 4.4 | 10.4 | 4.7 |
| 890.3 | 17.5 | 3.1 | 32.9 | 6.4 |
| 1589.8 | 18.3 | 4.1 | 36.4 | 3.0 |
| 5087.4 | 29.0 | 1.8 | 50.0 | 2.6 |
| 8902.9 | 45.3 | 2.0 | 51.7 | 2.9 |
| 15898.0 | 47.9 | 3.5 | 59.3 | 6.5 |
|  |  |  |  |  |
| Cry1A.105 protein_SR Pooled_Strain | | | | |
| Concentration (ng/cm^2^) | Corrected mortality (%) | SE | % Growth | SE |
| 0.0 | 0.0 | 0.0 | 0.0 | 0.3 |
| 1.6 | 12.5 | 1.0 | 26.3 | 1.0 |
| 5.1 | 32.8 | 0.8 | 35.5 | 1.3 |
| 15.9 | 46.9 | 1.4 | 51.3 | 0.3 |
| 50.9 | 62.5 | 1.7 | 61.8 | 0.8 |
| 159.0 | 71.9 | 1.2 | 76.1 | 0.3 |
| 508.7 | 78.9 | 1.1 | 83.6 | 0.3 |
| 1589.8 | 88.3 | 1.4 | 88.7 | 0.4 |
| 5087.4 | 96.9 | 1.2 | 96.4 | 0.2 |
|  |  |  |  |  |
|  |  |  |  |  |

| Cry2Ab2 protein_SS Strain | | | | |
| --- | --- | --- | --- | --- |
| Concentration (ng/cm^2^) | Corrected mortality (%) | SE | % Growth | SE |
| 0.0 | 0.0 | 0.0 | 0.0 | 0.0 |
| 0.5 | 7.8 | 3.5 | 27.3 | 2.9 |
| 1.6 | 20.3 | 2.5 | 45.7 | 2.1 |
| 5.1 | 39.1 | 2.8 | 57.7 | 3.9 |
| 15.9 | 60.3 | 2.7 | 61.9 | 3.7 |
| 50.9 | 68.8 | 2.1 | 69.3 | 3.8 |
| 159.0 | 75.0 | 2.3 | 75.3 | 2.6 |
| 508.7 | 85.9 | 2.5 | 85.7 | 3.9 |
| 1589.8 | 95.2 | 2.6 | 93.1 | 1.7 |
| 2861.6 | 100.0 | 0.0 | 100.0 | 0.0 |
| Cry2Ab2 protein_RR Strain | | | | |
| Concentration (ng/cm^2^) | Corrected mortality (%) | SE | % Growth | SE |
| 0.0 | 0.0 | 0.0 | 0.0 | 0.0 |
| 5.1 | 10.9 | 3.5 | 39.7 | 4.9 |
| 15.9 | 23.4 | 3.3 | 48.2 | 4.1 |
| 50.9 | 42.2 | 2.9 | 64.0 | 2.7 |
| 159.0 | 57.1 | 2.9 | 67.0 | 3.2 |
| 508.7 | 64.1 | 2.7 | 69.7 | 3.7 |
| 1589.8 | 77.4 | 2.6 | 73.3 | 2.2 |
| 4976.1 | 81.3 | 3.1 | 84.5 | 2.2 |
|  |  |  |  |  |
| Cry2Ab2 protein_SR Pooled Strain | | | | |
| Concentration (ng/cm^2^) | Corrected mortality (%) | SE | % Growth | SE |
| 0.0 | 0.0 | 0.0 | 0.0 | 0.0 |
| 0.5 | 3.1 | 1.2 | 25.5 | 5.4 |
| 1.6 | 13.3 | 1.4 | 36.8 | 4.7 |
| 5.1 | 15.6 | 1.6 | 46.0 | 3.0 |
| 15.9 | 32.0 | 1.4 | 53.9 | 2.3 |
| 50.9 | 64.1 | 1.0 | 63.4 | 2.3 |
| 159.0 | 68.8 | 0.6 | 83.4 | 4.4 |
| 508.7 | 71.1 | 1.1 | 91.5 | 2.0 |
| 1589.8 | 87.5 | 0.8 | 93.7 | 2.0 |
| 2861.6 | 99.2 | 0.0 | 95.5 | 1.2 |
