# Supplementary material for: Cross-Resistance between Cry1 Proteins in Fall Armyworm (Spodoptera frugiperda) May Affect the Durability of Current Pyramided Bt Maize Hybrids in Brazil
Source: PLoS One. 2015 Oct 16;10(10):e0140130. doi: 10.1371/journal.pone.0140130 (PMC4608726; doi:10.1371/journal.pone.0140130)
Supplement: S5 Table — (DOCX) [file pone.0140130.s005.docx]

**S5 Table.** Survival of *S. frugiperda* on leaf discs of different Bt and non-Bt maize plants.

| **ENTRY** | **REP** | **STRAIN** | **INFESTED** | **DEAD** | **LIVE** | **%MORTALITY** | **%SURVIVORSHIP** |
| --- | --- | --- | --- | --- | --- | --- | --- |
| TC 1507 (Cry1F) | 1 | RR | 12 | 0 | 12 | 0 | 100 |
| TC 1507 (Cry1F) | 2 | RR | 12 | 1 | 11 | 8 | 92 |
| TC 1507 (Cry1F) | 3 | RR | 12 | 0 | 12 | 0 | 100 |
| TC 1507 (Cry1F) | 4 | RR | 12 | 0 | 12 | 0 | 100 |
| TC 1507 (Cry1F) | 5 | RR | 12 | 0 | 12 | 0 | 100 |
| Non-Bt maize | 1 | RR | 12 | 0 | 12 | 0 | 100 |
| Non-Bt maize | 2 | RR | 12 | 0 | 12 | 0 | 100 |
| Non-Bt maize | 3 | RR | 12 | 0 | 12 | 0 | 100 |
| Non-Bt maize | 4 | RR | 12 | 0 | 12 | 0 | 100 |
| Non-Bt maize | 5 | RR | 12 | 0 | 12 | 0 | 100 |
| Cry1A.105 - single | 1 | RR | 12 | 0 | 12 | 0 | 100 |
| Cry1A.105 - single | 2 | RR | 12 | 4 | 8 | 33 | 67 |
| Cry1A.105 - single | 3 | RR | 12 | 3 | 9 | 25 | 75 |
| Cry1A.105 - single | 4 | RR | 12 | 0 | 12 | 0 | 100 |
| Cry1A.105 - single | 5 | RR | 12 | 5 | 7 | 42 | 58 |
| Cry2Ab2 - single | 1 | RR | 12 | 11 | 1 | 92 | 8 |
| Cry2Ab2 - single | 2 | RR | 12 | 12 | 0 | 100 | 0 |
| Cry2Ab2 - single | 3 | RR | 12 | 12 | 0 | 100 | 0 |
| Cry2Ab2 - single | 4 | RR | 12 | 12 | 0 | 100 | 0 |
| Cry2Ab2 - single | 5 | RR | 12 | 12 | 0 | 100 | 0 |
| MON 810 (Cry1Ab) | 1 | RR | 12 | 1 | 11 | 8 | 92 |
| MON 810 (Cry1Ab) | 2 | RR | 12 | 0 | 12 | 0 | 100 |
| MON 810 (Cry1Ab) | 3 | RR | 12 | 0 | 12 | 0 | 100 |
| MON 810 (Cry1Ab) | 4 | RR | 12 | 0 | 12 | 0 | 100 |
| MON 810 (Cry1Ab) | 5 | RR | 12 | 0 | 12 | 0 | 100 |
| TC 1507 (Cry1F) | 1 | SS | 12 | 12 | 0 | 100 | 0 |
| TC 1507 (Cry1F) | 2 | SS | 12 | 12 | 0 | 100 | 0 |
| TC 1507 (Cry1F) | 3 | SS | 12 | 12 | 0 | 100 | 0 |
| TC 1507 (Cry1F) | 4 | SS | 12 | 12 | 0 | 100 | 0 |
| TC 1507 (Cry1F) | 5 | SS | 12 | 12 | 0 | 100 | 0 |
| Cry2Ab2 - single | 1 | SS | 12 | 12 | 0 | 100 | 0 |
| Cry2Ab2 - single | 2 | SS | 12 | 12 | 0 | 100 | 0 |
| Cry2Ab2 - single | 3 | SS | 12 | 12 | 0 | 100 | 0 |
| Cry2Ab2 - single | 4 | SS | 12 | 12 | 0 | 100 | 0 |
| Cry2Ab2 - single | 5 | SS | 12 | 12 | 0 | 100 | 0 |
| Cry1A.105 - single | 1 | SS | 12 | 12 | 0 | 100 | 0 |
| Cry1A.105 - single | 2 | SS | 12 | 12 | 0 | 100 | 0 |
| Cry1A.105 - single | 3 | SS | 12 | 12 | 0 | 100 | 0 |
| Cry1A.105 - single | 4 | SS | 12 | 12 | 0 | 100 | 0 |
| Cry1A.105 - single | 5 | SS | 12 | 12 | 0 | 100 | 0 |
| MON 810 (Cry1Ab) | 1 | SS | 12 | 4 | 8 | 33 | 67 |
| MON 810 (Cry1Ab) | 2 | SS | 12 | 6 | 6 | 50 | 50 |
| MON 810 (Cry1Ab) | 3 | SS | 12 | 5 | 7 | 42 | 58 |
| MON 810 (Cry1Ab) | 4 | SS | 12 | 3 | 9 | 25 | 75 |
| MON 810 (Cry1Ab) | 5 | SS | 12 | 5 | 7 | 42 | 58 |
| Non-Bt maize | 1 | SS | 12 | 3 | 9 | 25 | 75 |
| Non-Bt maize | 2 | SS | 12 | 2 | 10 | 17 | 83 |
| Non-Bt maize | 3 | SS | 12 | 0 | 12 | 0 | 100 |
| Non-Bt maize | 4 | SS | 12 | 0 | 12 | 0 | 100 |
| Non-Bt maize | 5 | SS | 12 | 1 | 11 | 8 | 92 |

ENTRY: Corn material;

REP: Replication;

STRAIN: FAW colony;

INFESTED: Number of larvae infested by replication;

DEAD: Number of larvae dead after 5 days;

LIVE: Number of larvae live after 5 days;

| **ENTRY** | **REP** | **STRAIN** | **NLP** | **PL(g)** | **P_LARV (mg)** | **%_L1** | **%_L2** | **%_L3** |
| --- | --- | --- | --- | --- | --- | --- | --- | --- |
| TC 1507 (Cry1F) | 1 | RR | 12 | 0.1388 | 11.5667 | 0.0 | 8.3 | 91.7 |
| TC 1507 (Cry1F) | 2 | RR | 11 | 0.0807 | 7.3364 | 0.0 | 18.2 | 81.8 |
| TC 1507 (Cry1F) | 3 | RR | 12 | 0.1060 | 8.8333 | 0.0 | 0.0 | 100.0 |
| TC 1507 (Cry1F) | 4 | RR | 12 | 0.0966 | 8.0500 | 0.0 | 8.3 | 91.7 |
| TC 1507 (Cry1F) | 5 | RR | 12 | 0.0477 | 3.9750 | 0.0 | 0.0 | 100.0 |
| Non-Bt maize | 1 | RR | 12 | 0.0742 | 6.1833 | 0.0 | 0.0 | 100.0 |
| Non-Bt maize | 2 | RR | 12 | 0.0438 | 3.6500 | 0.0 | 0.0 | 100.0 |
| Non-Bt maize | 3 | RR | 11 | 0.0296 | 2.6909 | 0.0 | 8.3 | 91.7 |
| Non-Bt maize | 4 | RR | 12 | 0.2100 | 17.5000 | 0.0 | 0.0 | 100.0 |
| Non-Bt maize | 5 | RR | 12 | 0.0977 | 8.1417 | 0.0 | 0.0 | 100.0 |
| Cry1A.105 - single | 1 | RR | 12 | 0.0124 | 1.0333 | 0.0 | 100.0 | 0.0 |
| Cry1A.105 - single | 2 | RR | 8 | 0.0191 | 2.3875 | 0.0 | 75.0 | 37.5 |
| Cry1A.105 - single | 3 | RR | 7 | 0.0187 | 2.6714 | 0.0 | 77.8 | 22.2 |
| Cry1A.105 - single | 4 | RR | 11 | 0.0219 | 1.9909 | 0.0 | 100.0 | 0.0 |
| Cry1A.105 - single | 5 | RR | 7 | 0.0082 | 1.1714 | 0.0 | 100.0 | 0.0 |
| Cry2Ab2 - single | 1 | RR | 1 | 0.0000 | 0.0000 | 100.0 | 0.0 | 0.0 |
| Cry2Ab2 - single | 2 | RR | . | . | . | . | . | . |
| Cry2Ab2 - single | 3 | RR | . | . | . | . | . | . |
| Cry2Ab2 - single | 4 | RR | . | . | . | . | . | . |
| Cry2Ab2 - single | 5 | RR | . | . | . | . | . | . |
| MON 810 (Cry1Ab) | 1 | RR | 11 | 0.1018 | 9.2545 | 0.0 | 0.0 | 100.0 |
| MON 810 (Cry1Ab) | 2 | RR | 12 | 0.1041 | 8.6750 | 0.0 | 0.0 | 100.0 |
| MON 810 (Cry1Ab) | 3 | RR | 12 | 0.0890 | 7.4200 | 0.0 | 0.0 | 100.0 |
| MON 810 (Cry1Ab) | 4 | RR | 12 | 0.1064 | 8.8667 | 0.0 | 0.0 | 100.0 |
| MON 810 (Cry1Ab) | 5 | RR | 12 | 0.1173 | 9.7750 | 0.0 | 0.0 | 100.0 |
| TC 1507 (Cry1F) | 1 | SS | . | . | . | . | . | . |
| TC 1507 (Cry1F) | 2 | SS | . | . | . | . | . | . |
| TC 1507 (Cry1F) | 3 | SS | . | . | . | . | . | . |
| TC 1507 (Cry1F) | 4 | SS | . | . | . | . | . | . |
| TC 1507 (Cry1F) | 5 | SS | . | . | . | . | . | . |
| Cry2Ab2 - single | 1 | SS | . | . | . | . | . | . |
| Cry2Ab2 - single | 2 | SS | . | . | . | . | . | . |
| Cry2Ab2 - single | 3 | SS | . | . | . | . | . | . |
| Cry2Ab2 - single | 4 | SS | . | . | . | . | . | . |
| Cry2Ab2 - single | 5 | SS | . | . | . | . | . | . |
| Cry1A.105 - single | 1 | SS | . | . | . | . | . | . |
| Cry1A.105 - single | 2 | SS | . | . | . | . | . | . |
| Cry1A.105 - single | 3 | SS | . | . | . | . | . | . |
| Cry1A.105 - single | 4 | SS | . | . | . | . | . | . |
| Cry1A.105 - single | 5 | SS | . | . | . | . | . | . |
| MON 810 (Cry1Ab) | 1 | SS | 8 | 0.0303 | 3.7875 | 0.0 | 12.5 | 100.0 |
| MON 810 (Cry1Ab) | 2 | SS | 6 | 0.0221 | 3.6833 | 0.0 | 16.7 | 100.0 |
| MON 810 (Cry1Ab) | 3 | SS | 7 | 0.0261 | 3.7286 | 0.0 | 28.6 | 100.0 |
| MON 810 (Cry1Ab) | 4 | SS | 9 | 0.0499 | 5.5444 | 0.0 | 11.1 | 100.0 |
| MON 810 (Cry1Ab) | 5 | SS | 7 | 0.0334 | 4.7714 | 0.0 | 0.0 | 100.0 |
| Non-Bt maize | 1 | SS | 9 | 0.0484 | 5.3778 | 0.0 | 22.2 | 77.8 |
| Non-Bt maize | 2 | SS | 9 | 0.0646 | 7.1778 | 0.0 | 20.0 | 80.0 |
| Non-Bt maize | 3 | SS | 11 | 0.0893 | 8.1182 | 0.0 | 0.0 | 100.0 |
| Non-Bt maize | 4 | SS | 10 | 0.0797 | 7.9700 | 0.0 | 0.0 | 100.0 |
| Non-Bt maize | 5 | SS | 11 | 0.0828 | 7.5273 | 0.0 | 0.0 | 100.0 |

ENTRY: Corn material;

REP: Replicate;

STRAIN: FAW colony;

NLP: Number of larvae weighted;

PL(g): Weight of grouped larvae (g);

P_LARV (mg): Individual larva weight (mg);

%_L1, L2 and L3: Percent of larvae reaching 1^st^ , 2^nd^ or 3^rd^ instar after five days;
