# Supplementary material for: Cross-Resistance between Cry1 Proteins in Fall Armyworm (Spodoptera frugiperda) May Affect the Durability of Current Pyramided Bt Maize Hybrids in Brazil
Source: PLoS One. 2015 Oct 16;10(10):e0140130. doi: 10.1371/journal.pone.0140130 (PMC4608726; doi:10.1371/journal.pone.0140130)
Supplement: S6 Table — (DOCX) [file pone.0140130.s006.docx]

**S6 Table.** Comparison of fitness components of life stages of *S. frugiperda* strains reared on non-Bt corn.

| Strain | Time (days) | | | |
| --- | --- | --- | --- | --- |
|  | Egg | Larvae | Pupae | Egg-adult |
| RR | 2.9 | 15.8 | 10.0 | 28.7 |
| SR Pooled | 3.0 | 16.0 | 10.0 | 29.0 |
| SS | 3.0 | 15.7 | 8.9 | 27.6 |
| Strain | Standard Error | | | |
|  | Egg | Larvae | Pupae | Egg-adult |
| RR | 0.3 | 0.1 | 0.2 | 0.35 |
| SR Pooled | 0.6 | 0.2 | 0.2 | 0.5 |
| SS | 0.3 | 0.2 | 0.3 | 0.36 |
|  |  |  |  |  |
| Strain | Survival (%) | | | |
|  | Egg | Larvae | Pupae | Egg-adult |
| RR | 93.0 | 78.0 | 100 | 72.5 |
| SR Pooled | 95.5 | 93.5 | 100 | 87.7 |
| SS | 96.0 | 93.0 | 100 | 89.0 |
| Strain | Standard Error | | | |
|  | Egg | Larvae | Pupae | Egg-adult |
| SS | 0.7 | 2.0 | 0 | 2.7 |
| SR Pooled | 1.2 | 1.8 | 0 | 1.8 |
| RR | 0.7 | 3.5 | 0 | 3.4 |
