# Supplementary material for: Cross-Resistance between Cry1 Proteins in Fall Armyworm (Spodoptera frugiperda) May Affect the Durability of Current Pyramided Bt Maize Hybrids in Brazil
Source: PLoS One. 2015 Oct 16;10(10):e0140130. doi: 10.1371/journal.pone.0140130 (PMC4608726; doi:10.1371/journal.pone.0140130)
Supplement: S7 Table — (DOCX) [file pone.0140130.s007.docx]

**S7 Table.** Comparison of fitness cost components of females of *S. frugiperda* strains reared on non-Bt corn.

| Female longevity (days) | | Standard Error |
| --- | --- | --- |
| RR_strain | 14.3 | 0.5 |
| SR Pooled_ strain | 13.5 | 0.6 |
| SS_strain | 14.1 | 0.6 |
|  |  |  |
| Oviposition (days) | | Standard Error |
| RR_strain | 7.4 | 0.4 |
| SR Pooled_strain | 7.5 | 0.5 |
| SS_strain | 8.1 | 0.5 |
|  |  |  |
| Number of eggs/female | | Standard Error |
| RR_strain | 1573 | 99 |
| SR Pooled_strain | 1815 | 100 |
| SS_strain | 1869 | 139 |
|  |  |  |
| Eggs/female/day | | Standard Error |
| RR_strain | 180 | 12.5 |
| SR Pooled_strain | 232 | 11 |
| SS_strain | 250 | 19.3 |
