# Supplementary material for: Cross-Resistance between Cry1 Proteins in Fall Armyworm (Spodoptera frugiperda) May Affect the Durability of Current Pyramided Bt Maize Hybrids in Brazil
Source: PLoS One. 2015 Oct 16;10(10):e0140130. doi: 10.1371/journal.pone.0140130 (PMC4608726; doi:10.1371/journal.pone.0140130)
Supplement: S8 Table — (DOCX) [file pone.0140130.s008.docx]

**Table S8.** Fertility life table parameters of *S. frugiperda* strains fed on non-Bt maize.

| Strain | Number  female | Age of  female | Number  of eggs | Viability  total cycle | Sex  ratio |
| --- | --- | --- | --- | --- | --- |
| RR | 1 | 32.5 | 245 | 0.61 | 0.46 |
| RR | 1 | 33.5 | 386 | 0.61 | 0.46 |
| RR | 1 | 34.5 | 188 | 0.61 | 0.46 |
| RR | 1 | 35.5 | 132 | 0.61 | 0.46 |
| RR | 1 | 36.5 | 127 | 0.61 | 0.46 |
| RR | 1 | 37.5 | 108 | 0.61 | 0.46 |
| RR | 1 | 38.5 | 89 | 0.61 | 0.46 |
| RR | 2 | 32.5 | 624 | 0.61 | 0.46 |
| RR | 2 | 33.5 | 475 | 0.61 | 0.46 |
| RR | 2 | 34.5 | 203 | 0.61 | 0.46 |
| RR | 2 | 35.5 | 175 | 0.61 | 0.46 |
| RR | 2 | 36.5 | 60 | 0.61 | 0.46 |
| RR | 2 | 37.5 | 176 | 0.61 | 0.46 |
| RR | 2 | 38.5 | 101 | 0.61 | 0.46 |
| RR | 3 | 32.5 | 238 | 0.61 | 0.46 |
| RR | 3 | 33.5 | 456 | 0.61 | 0.46 |
| RR | 3 | 34.5 | 96 | 0.61 | 0.46 |
| RR | 3 | 35.5 | 198 | 0.61 | 0.46 |
| RR | 3 | 36.5 | 296 | 0.61 | 0.46 |
| RR | 3 | 37.5 | 271 | 0.61 | 0.46 |
| RR | 3 | 38.5 | 639 | 0.61 | 0.46 |
| RR | 3 | 39.5 | 232 | 0.61 | 0.46 |
| RR | 3 | 40.5 | 128 | 0.61 | 0.46 |
| RR | 4 | 32.5 | 412 | 0.61 | 0.46 |
| RR | 4 | 33.5 | 500 | 0.61 | 0.46 |
| RR | 4 | 34.5 | 175 | 0.61 | 0.46 |
| RR | 4 | 35.5 | 103 | 0.61 | 0.46 |
| RR | 4 | 36.5 | 80 | 0.61 | 0.46 |
| RR | 4 | 37.5 | 113 | 0.61 | 0.46 |
| RR | 4 | 38.5 | 200 | 0.61 | 0.46 |
| RR | 4 | 39.5 | 121 | 0.61 | 0.46 |
| RR | 5 | 32.5 | 777 | 0.61 | 0.46 |
| RR | 5 | 33.5 | 402 | 0.61 | 0.46 |
| RR | 5 | 34.5 | 202 | 0.61 | 0.46 |
| RR | 5 | 35.5 | 274 | 0.61 | 0.46 |
| RR | 5 | 36.5 | 157 | 0.61 | 0.46 |
| RR | 5 | 37.5 | 137 | 0.61 | 0.46 |
| RR | 5 | 38.5 | 84 | 0.61 | 0.46 |
| RR | 6 | 32.5 | 381 | 0.61 | 0.46 |
| RR | 6 | 33.5 | 218 | 0.61 | 0.46 |
| RR | 6 | 34.5 | 297 | 0.61 | 0.46 |
| RR | 6 | 35.5 | 162 | 0.61 | 0.46 |
| RR | 6 | 36.5 | 106 | 0.61 | 0.46 |
| RR | 6 | 37.5 | 80 | 0.61 | 0.46 |
| RR | 6 | 38.5 | 89 | 0.61 | 0.46 |
| RR | 7 | 32.5 | 205 | 0.61 | 0.46 |
| RR | 7 | 33.5 | 189 | 0.61 | 0.46 |
| RR | 7 | 34.5 | 118 | 0.61 | 0.46 |
| RR | 7 | 35.5 | 164 | 0.61 | 0.46 |
| RR | 7 | 36.5 | 38 | 0.61 | 0.46 |
| RR | 7 | 37.5 | 101 | 0.61 | 0.46 |
| RR | 7 | 38.5 | 80 | 0.61 | 0.46 |
| RR | 8 | 32.5 | 194 | 0.61 | 0.46 |
| RR | 8 | 33.5 | 139 | 0.61 | 0.46 |
| RR | 8 | 34.5 | 49 | 0.61 | 0.46 |
| RR | 8 | 35.5 | 105 | 0.61 | 0.46 |
| RR | 8 | 36.5 | 139 | 0.61 | 0.46 |
| RR | 8 | 37.5 | 100 | 0.61 | 0.46 |
| RR | 8 | 38.5 | 117 | 0.61 | 0.46 |
| RR | 8 | 39.5 | 58 | 0.61 | 0.46 |
| RR | 10 | 32.5 | 141 | 0.61 | 0.46 |
| RR | 10 | 33.5 | 191 | 0.61 | 0.46 |
| RR | 10 | 34.5 | 217 | 0.61 | 0.46 |
| RR | 10 | 35.5 | 139 | 0.61 | 0.46 |
| RR | 10 | 36.5 | 90 | 0.61 | 0.46 |
| RR | 10 | 37.5 | 78 | 0.61 | 0.46 |
| RR | 11 | 32.5 | 103 | 0.61 | 0.46 |
| RR | 11 | 33.5 | 532 | 0.61 | 0.46 |
| RR | 11 | 34.5 | 200 | 0.61 | 0.46 |
| RR | 11 | 35.5 | 191 | 0.61 | 0.46 |
| RR | 11 | 36.5 | 185 | 0.61 | 0.46 |
| RR | 11 | 37.5 | 79 | 0.61 | 0.46 |
| RR | 11 | 38.5 | 32 | 0.61 | 0.46 |
| RR | 12 | 32.5 | 139 | 0.61 | 0.46 |
| RR | 12 | 33.5 | 85 | 0.61 | 0.46 |
| RR | 12 | 34.5 | 244 | 0.61 | 0.46 |
| RR | 12 | 35.5 | 314 | 0.61 | 0.46 |
| RR | 12 | 36.5 | 153 | 0.61 | 0.46 |
| RR | 12 | 37.5 | 140 | 0.61 | 0.46 |
| RR | 12 | 38.5 | 135 | 0.61 | 0.46 |
| RR | 12 | 39.5 | 121 | 0.61 | 0.46 |
| RR | 13 | 32.5 | 76 | 0.61 | 0.46 |
| RR | 13 | 33.5 | 540 | 0.61 | 0.46 |
| RR | 13 | 34.5 | 461 | 0.61 | 0.46 |
| RR | 13 | 35.5 | 201 | 0.61 | 0.46 |
| RR | 13 | 36.5 | 146 | 0.61 | 0.46 |
| RR | 13 | 37.5 | 209 | 0.61 | 0.46 |
| RR | 13 | 38.5 | 38 | 0.61 | 0.46 |
| RR | 13 | 39.5 | 64 | 0.61 | 0.46 |
| RR | 14 | 32.5 | 101 | 0.61 | 0.46 |
| RR | 14 | 33.5 | 198 | 0.61 | 0.46 |
| RR | 14 | 34.5 | 298 | 0.61 | 0.46 |
| RR | 14 | 35.5 | 128 | 0.61 | 0.46 |
| RR | 14 | 36.5 | 397 | 0.61 | 0.46 |
| RR | 14 | 37.5 | 209 | 0.61 | 0.46 |
| RR | 14 | 38.5 | 256 | 0.61 | 0.46 |
| RR | 14 | 39.5 | 130 | 0.61 | 0.46 |
| RR | 14 | 40.5 | 75 | 0.61 | 0.46 |
| RR | 14 | 41.5 | 22 | 0.61 | 0.46 |
| RR | 15 | 32.5 | 516 | 0.61 | 0.46 |
| RR | 15 | 33.5 | 328 | 0.61 | 0.46 |
| RR | 15 | 34.5 | 631 | 0.61 | 0.46 |
| RR | 15 | 35.5 | 186 | 0.61 | 0.46 |
| RR | 15 | 36.5 | 51 | 0.61 | 0.46 |
| RR | 15 | 37.5 | 38 | 0.61 | 0.46 |
| RR | 15 | 38.5 | 40 | 0.61 | 0.46 |
| RR | 16 | 32.5 | 60 | 0.61 | 0.46 |
| RR | 16 | 33.5 | 740 | 0.61 | 0.46 |
| RR | 16 | 34.5 | 162 | 0.61 | 0.46 |
| RR | 16 | 35.5 | 320 | 0.61 | 0.46 |
| RR | 16 | 36.5 | 141 | 0.61 | 0.46 |
| RR | 16 | 37.5 | 57 | 0.61 | 0.46 |
| RR | 16 | 38.5 | 63 | 0.61 | 0.46 |
| RR | 16 | 39.5 | 45 | 0.61 | 0.46 |
| RR | 17 | 32.5 | 320 | 0.61 | 0.46 |
| RR | 17 | 33.5 | 94 | 0.61 | 0.46 |
| RR | 17 | 34.5 | 827 | 0.61 | 0.46 |
| RR | 17 | 35.5 | 311 | 0.61 | 0.46 |
| RR | 17 | 36.5 | 278 | 0.61 | 0.46 |
| RR | 17 | 37.5 | 50 | 0.61 | 0.46 |
| RR | 17 | 38.5 | 142 | 0.61 | 0.46 |
| RR | 19 | 32.5 | 520 | 0.61 | 0.46 |
| RR | 19 | 33.5 | 131 | 0.61 | 0.46 |
| RR | 19 | 34.5 | 250 | 0.61 | 0.46 |
| RR | 19 | 35.5 | 235 | 0.61 | 0.46 |
| RR | 19 | 36.5 | 128 | 0.61 | 0.46 |
| RR | 19 | 37.5 | 212 | 0.61 | 0.46 |
| RR | 19 | 38.5 | 100 | 0.61 | 0.46 |
| RR | 19 | 39.5 | 56 | 0.61 | 0.46 |
| RR | 20 | 32.5 | 420 | 0.61 | 0.46 |
| RR | 20 | 33.5 | 97 | 0.61 | 0.46 |
| RR | 20 | 34.5 | 179 | 0.61 | 0.46 |
| RR | 20 | 35.5 | 144 | 0.61 | 0.46 |
| RR | 20 | 36.5 | 105 | 0.61 | 0.46 |
| RR | 20 | 37.5 | 230 | 0.61 | 0.46 |
| RR | 20 | 38.5 | 127 | 0.61 | 0.46 |
| RR | 20 | 39.5 | 100 | 0.61 | 0.46 |
| RR | 20 | 40.5 | 28 | 0.61 | 0.46 |
| RR | 21 | 32.5 | 211 | 0.61 | 0.46 |
| RR | 21 | 33.5 | 141 | 0.61 | 0.46 |
| RR | 21 | 34.5 | 253 | 0.61 | 0.46 |
| RR | 21 | 35.5 | 250 | 0.61 | 0.46 |
| RR | 21 | 36.5 | 187 | 0.61 | 0.46 |
| RR | 21 | 37.5 | 156 | 0.61 | 0.46 |
| RR | 21 | 38.5 | 17 | 0.61 | 0.46 |
| RR | 21 | 39.5 | 100 | 0.61 | 0.46 |
| SS | 1 | 32.5 | 292 | 0.83 | 0.48 |
| SS | 1 | 33.5 | 113 | 0.83 | 0.48 |
| SS | 1 | 34.5 | 210 | 0.83 | 0.48 |
| SS | 1 | 35.5 | 311 | 0.83 | 0.48 |
| SS | 1 | 36.5 | 215 | 0.83 | 0.48 |
| SS | 1 | 37.5 | 312 | 0.83 | 0.48 |
| SS | 1 | 38.5 | 256 | 0.83 | 0.48 |
| SS | 1 | 39.5 | 120 | 0.83 | 0.48 |
| SS | 1 | 40.5 | 135 | 0.83 | 0.48 |
| SS | 2 | 32.5 | 275 | 0.83 | 0.48 |
| SS | 2 | 33.5 | 196 | 0.83 | 0.48 |
| SS | 2 | 34.5 | 149 | 0.83 | 0.48 |
| SS | 2 | 35.5 | 282 | 0.83 | 0.48 |
| SS | 2 | 36.5 | 211 | 0.83 | 0.48 |
| SS | 2 | 37.5 | 315 | 0.83 | 0.48 |
| SS | 2 | 38.5 | 200 | 0.83 | 0.48 |
| SS | 2 | 39.5 | 165 | 0.83 | 0.48 |
| SS | 2 | 40.5 | 121 | 0.83 | 0.48 |
| SS | 3 | 32.5 | 308 | 0.83 | 0.48 |
| SS | 3 | 33.5 | 189 | 0.83 | 0.48 |
| SS | 3 | 34.5 | 142 | 0.83 | 0.48 |
| SS | 3 | 35.5 | 182 | 0.83 | 0.48 |
| SS | 3 | 36.5 | 195 | 0.83 | 0.48 |
| SS | 3 | 37.5 | 211 | 0.83 | 0.48 |
| SS | 3 | 38.5 | 191 | 0.83 | 0.48 |
| SS | 4 | 32.5 | 210 | 0.83 | 0.48 |
| SS | 4 | 33.5 | 191 | 0.83 | 0.48 |
| SS | 4 | 34.5 | 200 | 0.83 | 0.48 |
| SS | 4 | 35.5 | 196 | 0.83 | 0.48 |
| SS | 4 | 36.5 | 175 | 0.83 | 0.48 |
| SS | 4 | 37.5 | 183 | 0.83 | 0.48 |
| SS | 4 | 38.5 | 121 | 0.83 | 0.48 |
| SS | 4 | 39.5 | 90 | 0.83 | 0.48 |
| SS | 4 | 40.5 | 56 | 0.83 | 0.48 |
| SS | 5 | 32.5 | 312 | 0.83 | 0.48 |
| SS | 5 | 33.5 | 428 | 0.83 | 0.48 |
| SS | 5 | 34.5 | 191 | 0.83 | 0.48 |
| SS | 5 | 35.5 | 112 | 0.83 | 0.48 |
| SS | 5 | 36.5 | 250 | 0.83 | 0.48 |
| SS | 5 | 37.5 | 200 | 0.83 | 0.48 |
| SS | 5 | 38.5 | 160 | 0.83 | 0.48 |
| SS | 5 | 39.5 | 112 | 0.83 | 0.48 |
| SS | 6 | 32.5 | 507 | 0.83 | 0.48 |
| SS | 6 | 33.5 | 312 | 0.83 | 0.48 |
| SS | 6 | 34.5 | 591 | 0.83 | 0.48 |
| SS | 6 | 35.5 | 420 | 0.83 | 0.48 |
| SS | 6 | 36.5 | 278 | 0.83 | 0.48 |
| SS | 6 | 37.5 | 112 | 0.83 | 0.48 |
| SS | 6 | 38.5 | 90 | 0.83 | 0.48 |
| SS | 6 | 39.5 | 95 | 0.83 | 0.48 |
| SS | 7 | 32.5 | 378 | 0.83 | 0.48 |
| SS | 7 | 33.5 | 163 | 0.83 | 0.48 |
| SS | 7 | 34.5 | 164 | 0.83 | 0.48 |
| SS | 7 | 35.5 | 200 | 0.83 | 0.48 |
| SS | 7 | 36.5 | 128 | 0.83 | 0.48 |
| SS | 7 | 37.5 | 135 | 0.83 | 0.48 |
| SS | 7 | 38.5 | 97 | 0.83 | 0.48 |
| SS | 7 | 39.5 | 85 | 0.83 | 0.48 |
| SS | 7 | 40.5 | 56 | 0.83 | 0.48 |
| SS | 7 | 41.5 | 31 | 0.83 | 0.48 |
| SS | 9 | 32.5 | 175 | 0.83 | 0.48 |
| SS | 9 | 33.5 | 202 | 0.83 | 0.48 |
| SS | 9 | 34.5 | 144 | 0.83 | 0.48 |
| SS | 9 | 35.5 | 157 | 0.83 | 0.48 |
| SS | 9 | 36.5 | 200 | 0.83 | 0.48 |
| SS | 9 | 37.5 | 135 | 0.83 | 0.48 |
| SS | 9 | 38.5 | 121 | 0.83 | 0.48 |
| SS | 9 | 39.5 | 85 | 0.83 | 0.48 |
| SS | 12 | 32.5 | 310 | 0.83 | 0.48 |
| SS | 12 | 33.5 | 412 | 0.83 | 0.48 |
| SS | 12 | 34.5 | 315 | 0.83 | 0.48 |
| SS | 12 | 35.5 | 400 | 0.83 | 0.48 |
| SS | 12 | 36.5 | 617 | 0.83 | 0.48 |
| SS | 12 | 37.5 | 551 | 0.83 | 0.48 |
| SS | 12 | 38.5 | 322 | 0.83 | 0.48 |
| SS | 12 | 39.5 | 285 | 0.83 | 0.48 |
| SS | 12 | 40.5 | 300 | 0.83 | 0.48 |
| SS | 13 | 32.5 | 192 | 0.83 | 0.48 |
| SS | 13 | 33.5 | 208 | 0.83 | 0.48 |
| SS | 13 | 34.5 | 315 | 0.83 | 0.48 |
| SS | 13 | 35.5 | 377 | 0.83 | 0.48 |
| SS | 13 | 36.5 | 154 | 0.83 | 0.48 |
| SS | 13 | 37.5 | 124 | 0.83 | 0.48 |
| SS | 13 | 38.5 | 100 | 0.83 | 0.48 |
| SS | 13 | 39.5 | 85 | 0.83 | 0.48 |
| SS | 13 | 40.5 | 96 | 0.83 | 0.48 |
| SS | 14 | 32.5 | 155 | 0.83 | 0.48 |
| SS | 14 | 33.5 | 485 | 0.83 | 0.48 |
| SS | 14 | 34.5 | 339 | 0.83 | 0.48 |
| SS | 14 | 35.5 | 295 | 0.83 | 0.48 |
| SS | 14 | 36.5 | 138 | 0.83 | 0.48 |
| SS | 14 | 37.5 | 186 | 0.83 | 0.48 |
| SS | 14 | 38.5 | 75 | 0.83 | 0.48 |
| SS | 14 | 39.5 | 63 | 0.83 | 0.48 |
| SS | 15 | 32.5 | 260 | 0.83 | 0.48 |
| SS | 15 | 33.5 | 317 | 0.83 | 0.48 |
| SS | 15 | 34.5 | 300 | 0.83 | 0.48 |
| SS | 15 | 35.5 | 283 | 0.83 | 0.48 |
| SS | 15 | 36.5 | 200 | 0.83 | 0.48 |
| SS | 15 | 37.5 | 195 | 0.83 | 0.48 |
| SS | 15 | 38.5 | 211 | 0.83 | 0.48 |
| SS | 15 | 39.5 | 136 | 0.83 | 0.48 |
| SS | 16 | 32.5 | 200 | 0.83 | 0.48 |
| SS | 16 | 33.5 | 312 | 0.83 | 0.48 |
| SS | 16 | 34.5 | 185 | 0.83 | 0.48 |
| SS | 16 | 35.5 | 215 | 0.83 | 0.48 |
| SS | 16 | 36.5 | 200 | 0.83 | 0.48 |
| SS | 16 | 37.5 | 226 | 0.83 | 0.48 |
| SS | 16 | 38.5 | 191 | 0.83 | 0.48 |
| SS | 16 | 39.5 | 100 | 0.83 | 0.48 |
| SS | 16 | 40.5 | 81 | 0.83 | 0.48 |
| SS | 18 | 33.5 | 332 | 0.83 | 0.48 |
| SS | 18 | 34.5 | 200 | 0.83 | 0.48 |
| SS | 18 | 35.5 | 491 | 0.83 | 0.48 |
| SS | 18 | 36.5 | 235 | 0.83 | 0.48 |
| SS | 18 | 37.5 | 197 | 0.83 | 0.48 |
| SS | 18 | 38.5 | 211 | 0.83 | 0.48 |
| SS | 18 | 39.5 | 135 | 0.83 | 0.48 |
| SS | 18 | 40.5 | 98 | 0.83 | 0.48 |
| SS | 18 | 41.5 | 76 | 0.83 | 0.48 |
| SS | 20 | 32.5 | 188 | 0.83 | 0.48 |
| SS | 20 | 33.5 | 121 | 0.83 | 0.48 |
| SS | 20 | 34.5 | 135 | 0.83 | 0.48 |
| SS | 20 | 35.5 | 200 | 0.83 | 0.48 |
| SS | 20 | 36.5 | 186 | 0.83 | 0.48 |
| SS | 20 | 37.5 | 221 | 0.83 | 0.48 |
| SS | 20 | 38.5 | 193 | 0.83 | 0.48 |
| SS | 20 | 39.5 | 175 | 0.83 | 0.48 |
| SS | 20 | 40.5 | 131 | 0.83 | 0.48 |
| SR Pooled | 1 | 34.5 | 445 | 0.9 | 0.48 |
| SR Pooled | 1 | 35.5 | 200 | 0.9 | 0.48 |
| SR Pooled | 1 | 36.5 | 121 | 0.9 | 0.48 |
| SR Pooled | 1 | 37.5 | 66 | 0.9 | 0.48 |
| SR Pooled | 1 | 38.5 | 21 | 0.9 | 0.48 |
| SR Pooled | 1 | 39.5 | 30 | 0.9 | 0.48 |
| SR Pooled | 2 | 34.5 | 86 | 0.9 | 0.48 |
| SR Pooled | 2 | 35.5 | 95 | 0.9 | 0.48 |
| SR Pooled | 2 | 36.5 | 64 | 0.9 | 0.48 |
| SR Pooled | 2 | 37.5 | 532 | 0.9 | 0.48 |
| SR Pooled | 2 | 38.5 | 307 | 0.9 | 0.48 |
| SR Pooled | 2 | 39.5 | 186 | 0.9 | 0.48 |
| SR Pooled | 2 | 40.5 | 91 | 0.9 | 0.48 |
| SR Pooled | 2 | 41.5 | 101 | 0.9 | 0.48 |
| SR Pooled | 3 | 34.5 | 206 | 0.9 | 0.48 |
| SR Pooled | 3 | 35.5 | 383 | 0.9 | 0.48 |
| SR Pooled | 3 | 36.5 | 290 | 0.9 | 0.48 |
| SR Pooled | 3 | 37.5 | 113 | 0.9 | 0.48 |
| SR Pooled | 3 | 38.5 | 389 | 0.9 | 0.48 |
| SR Pooled | 3 | 39.5 | 176 | 0.9 | 0.48 |
| SR Pooled | 3 | 40.5 | 100 | 0.9 | 0.48 |
| SR Pooled | 4 | 34.5 | 233 | 0.9 | 0.48 |
| SR Pooled | 4 | 35.5 | 291 | 0.9 | 0.48 |
| SR Pooled | 4 | 36.5 | 75 | 0.9 | 0.48 |
| SR Pooled | 4 | 37.5 | 154 | 0.9 | 0.48 |
| SR Pooled | 4 | 38.5 | 181 | 0.9 | 0.48 |
| SR Pooled | 4 | 39.5 | 135 | 0.9 | 0.48 |
| SR Pooled | 4 | 40.5 | 117 | 0.9 | 0.48 |
| SR Pooled | 5 | 34.5 | 31 | 0.9 | 0.48 |
| SR Pooled | 5 | 35.5 | 42 | 0.9 | 0.48 |
| SR Pooled | 5 | 36.5 | 182 | 0.9 | 0.48 |
| SR Pooled | 5 | 37.5 | 202 | 0.9 | 0.48 |
| SR Pooled | 5 | 38.5 | 286 | 0.9 | 0.48 |
| SR Pooled | 5 | 39.5 | 204 | 0.9 | 0.48 |
| SR Pooled | 5 | 40.5 | 312 | 0.9 | 0.48 |
| SR Pooled | 5 | 41.5 | 215 | 0.9 | 0.48 |
| SR Pooled | 5 | 42.5 | 75 | 0.9 | 0.48 |
| SR Pooled | 6 | 34.5 | 110 | 0.9 | 0.48 |
| SR Pooled | 6 | 35.5 | 213 | 0.9 | 0.48 |
| SR Pooled | 6 | 36.5 | 191 | 0.9 | 0.48 |
| SR Pooled | 6 | 37.5 | 169 | 0.9 | 0.48 |
| SR Pooled | 6 | 38.5 | 28 | 0.9 | 0.48 |
| SR Pooled | 6 | 39.5 | 57 | 0.9 | 0.48 |
| SR Pooled | 6 | 40.5 | 63 | 0.9 | 0.48 |
| SR Pooled | 7 | 34.5 | 82 | 0.9 | 0.48 |
| SR Pooled | 7 | 35.5 | 221 | 0.9 | 0.48 |
| SR Pooled | 7 | 36.5 | 300 | 0.9 | 0.48 |
| SR Pooled | 7 | 37.5 | 286 | 0.9 | 0.48 |
| SR Pooled | 7 | 38.5 | 379 | 0.9 | 0.48 |
| SR Pooled | 7 | 39.5 | 250 | 0.9 | 0.48 |
| SR Pooled | 7 | 40.5 | 185 | 0.9 | 0.48 |
| SR Pooled | 7 | 41.5 | 111 | 0.9 | 0.48 |
| SR Pooled | 8 | 34.5 | 226 | 0.9 | 0.48 |
| SR Pooled | 8 | 35.5 | 374 | 0.9 | 0.48 |
| SR Pooled | 8 | 36.5 | 295 | 0.9 | 0.48 |
| SR Pooled | 8 | 37.5 | 163 | 0.9 | 0.48 |
| SR Pooled | 8 | 38.5 | 175 | 0.9 | 0.48 |
| SR Pooled | 8 | 39.5 | 30 | 0.9 | 0.48 |
| SR Pooled | 8 | 40.5 | 25 | 0.9 | 0.48 |
| SR Pooled | 8 | 41.5 | 41 | 0.9 | 0.48 |
| SR Pooled | 9 | 34.5 | 92 | 0.9 | 0.48 |
| SR Pooled | 9 | 35.5 | 352 | 0.9 | 0.48 |
| SR Pooled | 9 | 36.5 | 224 | 0.9 | 0.48 |
| SR Pooled | 9 | 37.5 | 187 | 0.9 | 0.48 |
| SR Pooled | 9 | 38.5 | 158 | 0.9 | 0.48 |
| SR Pooled | 9 | 39.5 | 291 | 0.9 | 0.48 |
| SR Pooled | 9 | 40.5 | 186 | 0.9 | 0.48 |
| SR Pooled | 9 | 41.5 | 87 | 0.9 | 0.48 |
| SR Pooled | 9 | 42.5 | 63 | 0.9 | 0.48 |
| SR Pooled | 10 | 34.5 | 24 | 0.9 | 0.48 |
| SR Pooled | 10 | 35.5 | 421 | 0.9 | 0.48 |
| SR Pooled | 10 | 36.5 | 160 | 0.9 | 0.48 |
| SR Pooled | 10 | 37.5 | 256 | 0.9 | 0.48 |
| SR Pooled | 10 | 38.5 | 135 | 0.9 | 0.48 |
| SR Pooled | 10 | 39.5 | 217 | 0.9 | 0.48 |
| SR Pooled | 10 | 40.5 | 100 | 0.9 | 0.48 |
| SR Pooled | 11 | 34.5 | 34 | 0.9 | 0.48 |
| SR Pooled | 11 | 35.5 | 838 | 0.9 | 0.48 |
| SR Pooled | 11 | 36.5 | 276 | 0.9 | 0.48 |
| SR Pooled | 11 | 37.5 | 110 | 0.9 | 0.48 |
| SR Pooled | 11 | 38.5 | 64 | 0.9 | 0.48 |
| SR Pooled | 11 | 39.5 | 127 | 0.9 | 0.48 |
| SR Pooled | 11 | 40.5 | 62 | 0.9 | 0.48 |
| SR Pooled | 11 | 41.5 | 41 | 0.9 | 0.48 |
| SR Pooled | 12 | 34.5 | 96 | 0.9 | 0.48 |
| SR Pooled | 12 | 35.5 | 404 | 0.9 | 0.48 |
| SR Pooled | 12 | 36.5 | 97 | 0.9 | 0.48 |
| SR Pooled | 12 | 37.5 | 177 | 0.9 | 0.48 |
| SR Pooled | 12 | 38.5 | 92 | 0.9 | 0.48 |
| SR Pooled | 12 | 39.5 | 74 | 0.9 | 0.48 |
| SR Pooled | 12 | 40.5 | 68 | 0.9 | 0.48 |
| SR Pooled | 13 | 34.5 | 241 | 0.9 | 0.48 |
| SR Pooled | 13 | 35.5 | 381 | 0.9 | 0.48 |
| SR Pooled | 13 | 36.5 | 273 | 0.9 | 0.48 |
| SR Pooled | 13 | 37.5 | 183 | 0.9 | 0.48 |
| SR Pooled | 13 | 38.5 | 176 | 0.9 | 0.48 |
| SR Pooled | 13 | 39.5 | 142 | 0.9 | 0.48 |
| SR Pooled | 13 | 40.5 | 211 | 0.9 | 0.48 |
| SR Pooled | 13 | 41.5 | 96 | 0.9 | 0.48 |
| SR Pooled | 14 | 34.5 | 578 | 0.9 | 0.48 |
| SR Pooled | 14 | 35.5 | 365 | 0.9 | 0.48 |
| SR Pooled | 14 | 36.5 | 81 | 0.9 | 0.48 |
| SR Pooled | 14 | 37.5 | 30 | 0.9 | 0.48 |
| SR Pooled | 14 | 38.5 | 22 | 0.9 | 0.48 |
| SR Pooled | 14 | 39.5 | 82 | 0.9 | 0.48 |
| SR Pooled | 15 | 34.5 | 302 | 0.9 | 0.48 |
| SR Pooled | 15 | 35.5 | 693 | 0.9 | 0.48 |
| SR Pooled | 15 | 36.5 | 115 | 0.9 | 0.48 |
| SR Pooled | 15 | 37.5 | 104 | 0.9 | 0.48 |
| SR Pooled | 15 | 38.5 | 84 | 0.9 | 0.48 |
| SR Pooled | 15 | 39.5 | 130 | 0.9 | 0.48 |
| SR Pooled | 15 | 40.5 | 52 | 0.9 | 0.48 |
| SR Pooled | 16 | 34.5 | 28 | 0.9 | 0.48 |
| SR Pooled | 16 | 35.5 | 229 | 0.9 | 0.48 |
| SR Pooled | 16 | 36.5 | 205 | 0.9 | 0.48 |
| SR Pooled | 16 | 37.5 | 311 | 0.9 | 0.48 |
| SR Pooled | 16 | 38.5 | 112 | 0.9 | 0.48 |
| SR Pooled | 16 | 39.5 | 95 | 0.9 | 0.48 |
| SR Pooled | 18 | 34.5 | 618 | 0.9 | 0.48 |
| SR Pooled | 18 | 35.5 | 302 | 0.9 | 0.48 |
| SR Pooled | 18 | 36.5 | 244 | 0.9 | 0.48 |
| SR Pooled | 18 | 37.5 | 300 | 0.9 | 0.48 |
| SR Pooled | 18 | 38.5 | 166 | 0.9 | 0.48 |
| SR Pooled | 18 | 39.5 | 89 | 0.9 | 0.48 |
| SR Pooled | 18 | 40.5 | 136 | 0.9 | 0.48 |
| SR Pooled | 18 | 41.5 | 215 | 0.9 | 0.48 |
| SR Pooled | 19 | 34.5 | 41 | 0.9 | 0.48 |
| SR Pooled | 19 | 35.5 | 321 | 0.9 | 0.48 |
| SR Pooled | 19 | 36.5 | 223 | 0.9 | 0.48 |
| SR Pooled | 19 | 37.5 | 197 | 0.9 | 0.48 |
| SR Pooled | 19 | 38.5 | 100 | 0.9 | 0.48 |
| SR Pooled | 19 | 39.5 | 215 | 0.9 | 0.48 |
| SR Pooled | 19 | 40.5 | 78 | 0.9 | 0.48 |
| SR Pooled | 20 | 34.5 | 70 | 0.9 | 0.48 |
| SR Pooled | 20 | 35.5 | 542 | 0.9 | 0.48 |
| SR Pooled | 20 | 36.5 | 266 | 0.9 | 0.48 |
| SR Pooled | 20 | 37.5 | 391 | 0.9 | 0.48 |
| SR Pooled | 20 | 38.5 | 101 | 0.9 | 0.48 |
| SR Pooled | 20 | 39.5 | 100 | 0.9 | 0.48 |
| SR Pooled | 20 | 40.5 | 65 | 0.9 | 0.48 |
| SR Pooled | 20 | 41.5 | 41 | 0.9 | 0.48 |
| SR Pooled | 20 | 42.5 | 30 | 0.9 | 0.48 |
| SR Pooled | 21 | 34.5 | 321 | 0.9 | 0.48 |
| SR Pooled | 21 | 35.5 | 200 | 0.9 | 0.48 |
| SR Pooled | 21 | 36.5 | 354 | 0.9 | 0.48 |
| SR Pooled | 21 | 37.5 | 96 | 0.9 | 0.48 |
| SR Pooled | 21 | 38.5 | 112 | 0.9 | 0.48 |
| SR Pooled | 21 | 39.5 | 126 | 0.9 | 0.48 |
| SR Pooled | 21 | 40.5 | 99 | 0.9 | 0.48 |
| SR Pooled | 21 | 41.5 | 40 | 0.9 | 0.48 |
| SR Pooled | 21 | 42.5 | 52 | 0.9 | 0.48 |
| SR Pooled | 21 | 43.5 | 35 | 0.9 | 0.48 |
